# Cuprorivaite/hardystonite/alginate composite hydrogel with thermionic effect for the treatment of peri-implant lesion

**DOI:** 10.1093/rb/rbae028

**Published:** 2024-03-21

**Authors:** Yiru Xia, Zhaowenbin Zhang, Kecong Zhou, Zhikai Lin, Rong Shu, Yuze Xu, Zhen Zeng, Jiang Chang, Yufeng Xie

**Affiliations:** Department of Periodontology, Shanghai Ninth People’s Hospital, Shanghai Jiao Tong University School of Medicine, Shanghai, China; College of Stomatology, Shanghai Jiao Tong University, National Center for Stomatology, National Clinical Research Center for Oral Diseases, Shanghai Key Laboratory of Stomatology, Shanghai Research Institute of Stomatology, Shanghai, China; Joint Centre of Translational Medicine, the First Affiliated Hospital of Wenzhou Medical University, Wenzhou 325000, China; Zhejiang Engineering Research Center for Tissue Repair Materials, Wenzhou Institute, University of Chinese Academy of Sciences, Wenzhou 325000, China; State Key Laboratory of High-Performance Ceramics and Superfine Microstructure, Shanghai Institute of Ceramics, Chinese Academy of Sciences, Shanghai 200050, China; Department of Periodontology, Shanghai Ninth People’s Hospital, Shanghai Jiao Tong University School of Medicine, Shanghai, China; College of Stomatology, Shanghai Jiao Tong University, National Center for Stomatology, National Clinical Research Center for Oral Diseases, Shanghai Key Laboratory of Stomatology, Shanghai Research Institute of Stomatology, Shanghai, China; Department of Periodontology, Shanghai Ninth People’s Hospital, Shanghai Jiao Tong University School of Medicine, Shanghai, China; College of Stomatology, Shanghai Jiao Tong University, National Center for Stomatology, National Clinical Research Center for Oral Diseases, Shanghai Key Laboratory of Stomatology, Shanghai Research Institute of Stomatology, Shanghai, China; Department of Periodontology, Shanghai Ninth People’s Hospital, Shanghai Jiao Tong University School of Medicine, Shanghai, China; College of Stomatology, Shanghai Jiao Tong University, National Center for Stomatology, National Clinical Research Center for Oral Diseases, Shanghai Key Laboratory of Stomatology, Shanghai Research Institute of Stomatology, Shanghai, China; Joint Centre of Translational Medicine, the First Affiliated Hospital of Wenzhou Medical University, Wenzhou 325000, China; Zhejiang Engineering Research Center for Tissue Repair Materials, Wenzhou Institute, University of Chinese Academy of Sciences, Wenzhou 325000, China; State Key Laboratory of High-Performance Ceramics and Superfine Microstructure, Shanghai Institute of Ceramics, Chinese Academy of Sciences, Shanghai 200050, China; Joint Centre of Translational Medicine, the First Affiliated Hospital of Wenzhou Medical University, Wenzhou 325000, China; Zhejiang Engineering Research Center for Tissue Repair Materials, Wenzhou Institute, University of Chinese Academy of Sciences, Wenzhou 325000, China; State Key Laboratory of High-Performance Ceramics and Superfine Microstructure, Shanghai Institute of Ceramics, Chinese Academy of Sciences, Shanghai 200050, China; Key Laboratory of Rehabilitation Medicine in Sichuan Province, West China Hospital, Sichuan University, Chengdu, China; Joint Centre of Translational Medicine, the First Affiliated Hospital of Wenzhou Medical University, Wenzhou 325000, China; Zhejiang Engineering Research Center for Tissue Repair Materials, Wenzhou Institute, University of Chinese Academy of Sciences, Wenzhou 325000, China; State Key Laboratory of High-Performance Ceramics and Superfine Microstructure, Shanghai Institute of Ceramics, Chinese Academy of Sciences, Shanghai 200050, China; Department of Periodontology, Shanghai Ninth People’s Hospital, Shanghai Jiao Tong University School of Medicine, Shanghai, China; College of Stomatology, Shanghai Jiao Tong University, National Center for Stomatology, National Clinical Research Center for Oral Diseases, Shanghai Key Laboratory of Stomatology, Shanghai Research Institute of Stomatology, Shanghai, China; Department of Periodontology, Shanghai Stomatological Hospital & School of Stomatology, Fudan University, Shanghai, China; Shanghai Key Laboratory of Craniomaxillofacial Development and Diseases, Fudan University, Shanghai, China

**Keywords:** bioceramics, composite hydrogel, thermionic effect, peri-implant lesion

## Abstract

Peri-implant lesion is a grave condition afflicting numerous indi-viduals with dental implants. It results from persistent periodontal bacteria accumulation causing inflammation around the implant site, which can primarily lead to implant loosening and ultimately the implant loss. Early-stage peri-implant lesions exhibit symptoms akin to gum disease, including swelling, redness and bleeding of the gums surrounding the implant. These signs indicate infection and inflammation of the peri-implant tissues, which may result in bone loss and implant failure. To address this problem, a thermionic strategy was applied by designing a cuprorivaite–hardystonite bioceramic/alginate composite hydrogel with photothermal and Cu/Zn/Si multiple ions releasing property. This innovative approach creates a thermionic effect by the release of bioactive ions (Cu^2+^ and Zn^2+^ and ${\text{SiO}}_{3}^{2-}$) from the composite hydrogel and the mild heat environment though the photothermal effect of the composite hydrogel induced by near-infrared light irradiation. The most distinctive advantage of this thermionic effect is to substantially eliminate periodontal pathogenic bacteria and inhibit inflammation, while simultaneously enhance peri-implant osseointegration. This unique attribute renders the use of this composite hydrogel highly effective in significantly improving the survival rate of implants after intervention in peri-implant lesions, which is a clinical challenge in periodontics. This study reveals application potential of a new biomaterial-based approach for peri-implant lesion, as it not only eliminates the infection and inflammation, but also enhances the osteointegration of the dental implant, which provides theoretical insights and practical guidance to prevent and manage early-stage peri-implant lesion using bioactive functional materials.

## Graphical abstract

**Figure:**
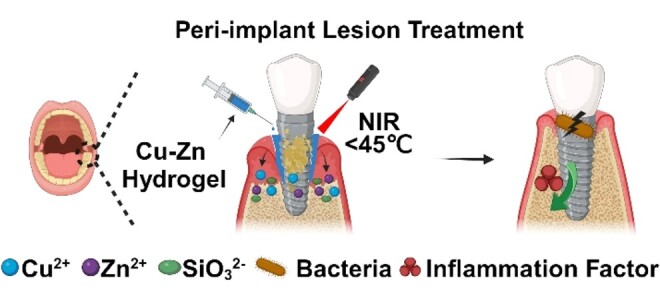

## Introduction

The oral cavity is an optimal breeding ground for bacteria in the human body due to its constant temperature of 37°C, ample nutrients (food residues) and moisture, which facilitates the accumulation of pathogenic bacteria and formation of dental plaque biofilms on tooth surfaces, ultimately leading to oral diseases [1, 2]. The “Global Oral Health Status Report” by the World Health Organization (2022) states that approximately 2 billion people globally suffer from permanent tooth decay, resulting in tooth loss that significantly impacts patients' daily lives [3]. Dental implants have been developed as artificial tooth roots, typically placed in the alveolar bone (AB) to restore both function and aesthetics to missing teeth [4]. However, the process of osseointegration, which is the direct contact and integration of bone tissue with the implant surface, usually takes 2–6 months after implantation [5]. Throughout this long period, periodontal pathogenic bacteria within the oral cavity persistently proliferate, accumulate around the implant site and often result in inflammation and finally the peri-implant lesion, which leads to bone resorption and the failure of implant osseointegration [6–8]. Presently, there is a lack of effective clinical treatments or managements for inflammation during implant osseointegration stage. Although clinicians may employ mechanical curettage to remove plaque and calculus from implant surfaces [6, 9], this approach overlooks the incomplete integration of bone during the initial implantation stage. Such mechanical intervention can disrupt the implant–bone connection, potentially resulting in a decreased implant survival rate of only 78.3% post-implantation [10]. Therefore, the development of novel therapeutic approaches that prevent peri-implant lesion by inhibiting inflammation and bacteria growth while promoting osseointegration is a challenge in oral implantology.

Efficiently eradicating periodontal pathogenic bacteria that infiltrate implants is a pivotal precondition for treating peri-implant lesion. Copper ions (Cu^2+^) and zinc ions (Zn^2+^) currently stand as prevalent and potent inorganic antibacterial agents, exhibiting broad-spectrum antibacterial efficacy [11, 12]. Cu^2+^ primarily penetrates bacterial structures by disrupting their outer membranes, influencing cellular metabolism and respiration, resulting in the loss of activity. Zn^2+^ predominantly binds to the negative charge on bacterial biofilms, altering their permeability and stability, ultimately disrupting bacterial reproductive functions [13, 14]. Notably, both Cu^2+^ ions alone and Zn^2+^ ions alone have found extensive use as additives in toothpaste and mouthwash to inhibit periodontal pathogenic bacteria, thereby aiding in the prevention of periodontitis [15]. Nonetheless, the synergistic effects of these two ions on periodontal pathogenic bacteria remain unknown. This lack of exploration may be due to the fact that the combination of these two ions at antibacterial concentrations of each single ion may result in higher toxicity, and reduced ion concentration may lead to lower antibacterial activity [16–19]. In addition to the antibacterial approach of metal ions, photothermal therapy (PTT) is also increasingly recognized as a highly effective strategy against bacterial infections [20, 21]. Extensive research has led to the development of photothermal materials capable of converting near-infrared (NIR) light energy into thermal energy. This resultant thermal energy not only inhibits the growth of drug-resistant bacteria but also disrupts the formation of bacterial biofilm structures, effectively exterminating bacteria without application of antibiotics [22]. However, the temperature required for photothermal antibacterial action tends to be relatively high (around 55–60°C) [18, 23], which can also potentially harm normal tissues and exacerbate inflammatory reactions [24].

Apart from the antibacterial activity of Cu^2+^ and Zn^2+^ ions, they also showed activity in enhancing tissue regeneration, in particular in combination with silicate (${\text{SiO}}_{3}^{2-}$) ions [25, 26]. Previous studies have shown that Cu-containing silicate bioceramics exhibit remarkable angiogenesis, whereas Zn-containing silicates have activity to inhibit inflammation [27, 28]. Furthermore, our previous study has revealed that the mild heating enhances the bioactivity of Cu ions released from silicate bioceramics, which suggests that this thermionic effect by the combination of mild heating and the ions-releasing biomaterials may further enhance the therapeutic effects of biomaterials [18, 20]. Moreover, the silicate bioceramics alone have also been wildly studied for their high osteogenic activity, especially in oral field [29, 30]. Inspired by these findings, we hypothesize that the thermionic effect of multiple-ion combination may be significantly higher than the bioactivity of each single ions and combination of mild heating with Cu–Zn-silicates may reveal dual-functional properties such as inhibiting inflammation and bacteria growth, and enhancing bone integration, and ultimately inhibit peri-implant lesion.

**Figure 1.**
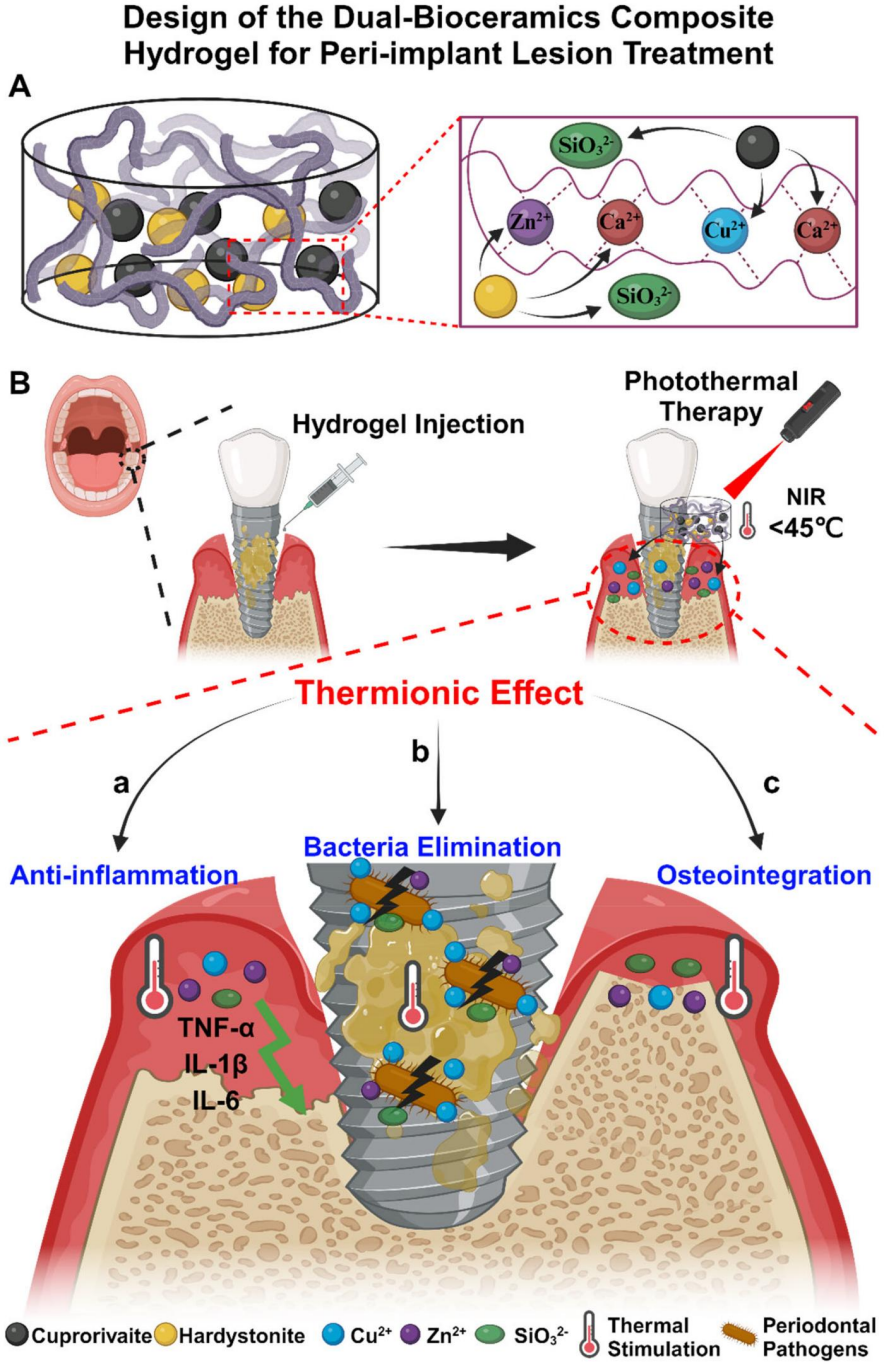
Design of the dual-bioceramics (cuprorivaite/hardystonite) composite hydrogel for peri-implant lesion treatment. (**A**) The composite hydrogel is formed by the triple-cross-linking of the alginate through Cu^2+^, Zn^2+^ and Ca^2+^ gradually released from cuprorivaite and hardystonite. (**B**) The composite hydrogel creates thermionic effect through low temperature photothermal irradiation and release of Cu^2+^, Zn^2+^ and ${\text{SiO}}_{3}^{2-}$ ions, which possesses anti-inflammation (a), bacteria elimination (b) and osteointegration (c) activities for the peri-implant lesion treatment.

To prove our hypotheses, we fabricated a dual-bioceramics/alginate composite hydrogel, which contains a Cu containing silicate bioceramic (cuprorivaite; CaCuSi_4_O_10_) and a Zn containing silicate bioceramic (hardystonite; Ca_2_ZnSi_2_O_7_). This design ensures the release of divalent ions such as Ca^2+^, Cu^2+^ and Zn^2+^ ions which crosslink with alginate molecules to form injectable hydrogel upon mixing the ceramics particles with alginate solution. Distinguished by remarkable photothermal properties and sustained release of bioactive Cu^2+^, Zn^2+^ and ${\text{SiO}}_{3}^{2-}$ ions, this composite hydrogel creates a distinct thermionic effect to simultaneously suppress inflammation, eliminate bacteria and enhance osteointegration (Figure 1). The biological implications of the thermionic effect were validated through *in vitro* cell experiments. Concurrently, an early-stage peri-implant lesion model induced by periodontal pathogenic bacteria following implantation was established to evaluate the diverse benefits of the composite hydrogel in eliminating bacterial infection, suppressing inflammation and enhancing osteointegration of the implant.

## Materials and methods

### Materials

Sodium alginate (A110570) and tetraethyl orthosilicate (T110593, TEOS) were bought from Aladdin Co., Ltd (Shanghai, China). Copper nitrate (10007916, Cu(NO_3_)_2_), zinc nitrate (80141318, Zn(NO_3_)_2_), calcium nitrate (80029062, Ca(NO_3_)_2_), sodium chloride (10019308, NaCl) and phosphate-buffered saline (72013560, PBS) buffer were bought from Solarbio Co., Ltd (Beijing, China). Dulbecco’s modified Eagle’s medium high glucose (A110570, DMEM), fetal bovine serum (G8001, FBS), penicillin/streptomycin (G4003) and cell counting kit-8 (G4103, CCK-8) were purchased from Servicebio Technology Co., Ltd (Wuhan, China).

### Preparation of cuprorivaite/hardystonite (CaCuSi_4_O_10_/Ca_2_ZnSi_2_O^−^) bioceramics composite hydrogels (Cu–Zn)

Three percentage sodium alginate solution was prepared by dissolving 3 g of sodium alginate in 100 ml of deionized water. Subsequently, 0.1 g of cuprorivaite bioceramic powder and 0.1 g of hardystonite bioceramic powder were individually weighed. The aforementioned powders were thoroughly mixed with 10 ml of the sodium alginate solution using a syringe and connecting tube, resulting in the formation of a composite hydrogel (Cu–Zn) containing 1% cuprorivaite and 1% hardystonite. Moreover, Control groups were established: a composite hydrogel consisting solely of 1% cuprorivaite (Cu), a composite hydrogel containing only 1% hardystonite (Zn) and a hydrogel devoid of any bioceramics (Control, crosslinking with calcium chloride). The gelation time of the Cu–Zn composite hydrogels was measured using a stopwatch. Cu hydrogels containing different concentrations of cuprorivaite (1% and 2%), Zn hydrogels containing different concentrations of hardystonite (1% and 2%), and a Control hydrogel cross-linking with calcium chloride were designed for comparison. Subsequently, the hydrogel samples were subjected to freeze-drying, and their surface morphology was examined through scanning electron microscopy (SEM, S-4800, Hitachi, Japan). In conjunction with an energy-dispersive X-ray spectroscopy (EDS) system, the elemental type and content within microscopic areas of the material were analyzed. The time-dependent fluidity and injectability of composite hydrogel were recorded through video camera. A rheometer (MCR 301; Anton Paar, Austria) was utilized to delve into the rheological characteristics of the hydrogels. Specifically, the correlation between the storage modulus (Gʹ) and the loss modulus (G″ʹ) was scrutinized at a frequency of 1 Hz. To assess the mechanical property of the hydrogels, a mechanical tester (INSTRON 5982) was employed. Initially, hydrogels with defined dimensions of 15 mm in height and diameter were fabricated. These hydrogels underwent uniaxial compression at a consistent strain rate of 1 mm/min. The compressive strength was gauged by the ultimate stress at the point of failure. Additionally, a thermal imaging camera (PM100D, Thorlabs GmbH, Munich, Germany) was engaged to monitor the temperature variations of the composite hydrogel when exposed to NIR laser irradiation (808 nm, 0.30 W/cm^2^). The ion release characteristics of the composite hydrogels were assessed as follows: distinct groups of composite hydrogels (1 ml each) were introduced into 10 ml of PBS buffer solution and subsequently incubated for varying durations (1st, 2nd, 3rd, 4th and 5th day). Following the incubation, the suspension was centrifuged at 4000 r/min for 10 min at predetermined intervals. The released medium was collected, and 10 ml of fresh PBS solution was introduced in place of the collected medium. Subsequently, the concentrations of Cu^2+^ and Zn^2+^ and ${\text{SiO}}_{3}^{2-}$ ions released from the hydrogel were quantified using an inductively coupled plasma atomic emission spectrometer (ICP-AES, Thermo Fisher X Series 2, USA).

### Antibacterial effect of composite hydrogel against *F. nucleatum*

The hydrogel samples and *Fusobacterium nucleatum* (ATCC 25586, Shanghai Key Laboratory of Stomatology, China) suspension in logarithmic growth phase (approximately 1 × 10^9^ CFU/ml) were combined in a sterile EP tube (5 ml), with a ratio of 1 ml hydrogel to 2 ml bacterial suspension. The distinct experimental groups were as follows: Blank group, Cu group, Zn group, Cu–Zn group and Cu–Zn + Laser group. Specifically, the Cu–Zn hydrogels of the Cu–Zn + Laser group were subjected to NIR light irradiation to maintain a photothermal temperature to 45°C. After 20 min, samples were placed in an anaerobic incubator and cultured for 6 h. Subsequently, 100 μl of bacterial solution was drawn for a 10-fold dilution. Fifty microliters of this diluted bacterial solution was smeared onto a blood plate, and colony counting was conducted following 3 days of anaerobic culture of *F. nucleatum* post-plating.

Furthermore, the effect of the hydrogel on the morphology of *F. nucleatum* was examined using SEM. Initially, 2 ml of bacterial solution in the logarithmic growth phase was mixed with 1 ml of distinct gel samples. The specific groups included the Blank group, Cu group, Zn group, Cu–Zn group and Cu–Zn + Laser group. Subsequently, the bacteria treated with the composite hydrogel were placed in an anaerobic incubator for 24 h. The bacterial solution was then centrifuged, the supernatant discarded and the bacterial pellets washed twice with sterile PBS. After centrifugation and discarding the PBS, the bacteria were fixed with 2.5% glutaraldehyde overnight at 4°C. A small amount of the fixed bacterial solution was spread on a coverslip coated with 0.1% polylysine, and after a 20-min interval, the coverslip was further fixed in 2.5% glutaraldehyde for an additional hour. Gradual dehydration was performed using varying concentrations of ethanol, starting from 30% and increasing in 10% increments up to 100%. Subsequently, the samples were air-dried in a clean bench and coated with gold before being examined using a SEM (JEOL JSM-6360LV, Japan).

### Analysis of rat bone marrow-derived mesenchymal stem cells viability *in vitro* affected by composite hydrogels

The impact of hydrogel materials on rat bone marrow-derived mesenchymal stem cells (rBMSCs) viability *in vitro* was investigated. The experimental groups consisted of the Blank group, Cu group, Zn group, Cu–Zn group and Cu–Zn + Laser group. Initially, in accordance with ISO 10993-5 standards, a hydrogel leach solution (1 ml of α-MEM with 10% FBS, v/v, at 37°C) was prepared and subjected to a 3-day incubation period. This solution was then filter-sterilized for subsequent cytotoxicity assay and evaluated using the CCK-8. rBMSCs were cultured in 96-well plates (Nest, USA) at a density of 1 × 10^4^ cells per well for 1 day. Subsequently, 100% leaching solution (100 μl/well) from each material group was added, and the cells were cultured for 1, 4 and 7 days. A volume of 10 μl of CCK-8 (Dojindo, Japan) was introduced to each well, and incubated in darkness for 2 h. Absorbance at 450 nm was measured using a microplate reader (Bio-Tek, USA). The values from negative control wells were averaged and considered to represent 100% cell viability. All other values were then averaged relative to their respective groups and compared to the negative control.

A 12-well plate was utilized to uniformly apply 1% weight hydrogel material. Subsequently, the plate was exposed to 405 nm ultraviolet light for 20 s. rBMSCs in logarithmic growth phase were enzymatically digested, and the cell density was adjusted to 1 × 10^5^ cells/ml. These cells were then inoculated onto the hydrogel and placed within a 37°C cell culture incubator. Following 24 h of incubation, a living-dead staining working solution, prepared according to the manufacturer's instructions, was added to the corresponding well plate. Incubation was performed in a 37°C, 5% CO_2_ cell culture incubator for 15 min. Fluorescent images of the cell groups were captured using a fluorescent microscope (Olympus, CKX53, Japan).

### Characterization of *in vitro* osteogenic activity for composite hydrogels

The procedure for inducing cell osteogenesis encompassed the following stages: a 12-well cell culture plate was chosen, and a hydrogel material weighing 1% of the total was spread across the plate. Before use, the plate was exposed to 405 nm ultraviolet light for 20 s. The second to third generation rBMSCs, demonstrating robust cell growth, were selected. These cells were subjected to an osteogenic induction medium (OriCell, HUXXC-90021, USA) consisting of 50 mg/ml ascorbic acid, 10 nmol/ml β-glycerophosphate sodium and 10 nmol/ml dexamethasone for the purpose of osteogenic induction culture.

To assess the expression of ALP, an alkaline phosphatase chromogenic kit (Beyotime, C3250S, China) was employed. Following 3 and 14 days of osteogenic induction and differentiation, the complete osteogenic differentiation medium in the small dish was aspirated, and the cells were gently washed with 1× PBS 2–3 times. A staining working solution was added to each sample to ensure complete coverage. Incubation was carried out at room temperature in the dark for 5 min. Afterward, the staining working solution was removed, and the cells were washed 1–2 times with distilled water to halt the color reaction. Subsequently, 2 ml of 1× PBS was added to each well, and the culture dish was positioned under an inverted microscope for ALP staining analysis and image capture.

For the detection of calcium deposition and calcified nodules in the cells of each group, an alizarin red S staining solution (Solarbio, G1450, China) was utilized. Following 3 and 14 days of osteogenesis induction and differentiation, the complete osteogenesis induction and differentiation medium was removed from the small dish. Gently washing the cells with 1× PBS for 2–3 times was performed. Thereafter, 2 ml of 4% paraformaldehyde solution was added to each well to fix the cells at room temperature for 30 min. The fixative was then aspirated, and the cells were washed gently with 1× PBS 2–3 times to ensure thorough washing of the fixative. A 2 ml Alizarin Red working solution was added to each well, allowing for staining at room temperature for 10 min. The cells were then washed gently with 1× PBS 2–3 times to effectively remove any excess staining solution. Following this, 2 ml of 1× PBS was added to each well, and the samples were placed under a microscope to observe the effects of osteogenic staining and capture images for subsequent analysis.

### Real-time PCR experiment

Upon completion of 14 days of osteogenic induction and differentiation, cellular RNA was extracted following the guidelines of the respective reagent manufacturer. Subsequently, cDNA was synthesized through reverse transcription, and the expression of pertinent genes was analyzed. The subsequent steps are succinctly elucidated as follows:

Cell lysis was accomplished using Trizol (Invitrogen, USA), with the addition of one-fifth volume of chloroform. The mixture was vigorously shaken and left at room temperature for a duration of 5 min. Centrifugation was performed at 12 000 r/min for 15 min at 4°C, and the upper aqueous phase was meticulously transferred into a new RNase-free EP tube. An equal volume of isopropanol was added, thoroughly mixed and left at room temperature for a period of 10 min. Further centrifugation was undertaken at 12 000 r/min for 10 min at 4°C. The supernatant was discarded, revealing a white precipitate at the base of the EP tube. Washing of the precipitate was conducted using 75% ethanol prepared with diethyl pyrocarbonate (DEPC) water, followed by centrifugation at 8000 r/min at 4°C for 5 min. After discarding the supernatant, the precipitate was allowed to air dry at room temperature for 10–20 min. Subsequently, the precipitate was dissolved using 15–20 μl of DEPC water. The RNA concentration and purity were measured, paving the way for the ensuing reverse transcription reaction.

For reverse transcription, the Prime Script™ RT reagent kit (Takara, Japan) was employed. To gauge gene expression, the SYBR^®^ Premix Ex Taq™ (Takara, Japan) was utilized, and the relative quantitative 2−△△Ct method was employed to compute the relative expression of the target gene in the experimental group as well as the Control group. The primer sequences were comprehensively exhibited in Supplementary Table S1.

### Establishment of rat model for peri-implant lesion and the hydrogel treatment

The experiment received approval from the Ethics Committee of the Ninth People's Hospital Affiliated with Shanghai Jiaotong University School of Medicine, under ethics number SH9H-2020-A129-1. The experimental animals were procured from Shanghai Xipuer-Bikay Laboratory Animal Co., Ltd. The customized titanium alloy implant, as illustrated in Supplementary Figure S1, was procured from Jiangsu Chuangying Medical Instrument Co., Ltd This implant exhibited an upper and lower configuration. The upper part featured a smooth neck structure with a diameter of 1.5 ± 0.5 mm and a height of 1.5 mm. In contrast, the lower part was characterized by a self-tapping tapered thread structure. Its surface underwent treatment through coarse sandblasting and acid etching (SLA), with a height measuring 1.5 mm. A total of 36 SPF male SD rats, aged 8 weeks, were meticulously selected and individually housed in separate cages. They had unrestricted access to water and were maintained under ambient conditions at a temperature of 20–22°C, following a light–dark cycle of 12:12 (lights on at 07:00, lights off at 19:00). The rats were assigned into randomized groups, comprising six rats in each group. General anesthesia was induced through intraperitoneal injection of 1.6% sultamate + 4% dexmedetomidine (3 ml/kg). Subsequently, SLA titanium alloy implants (3.0 × 1.5 mm) were surgically inserted in front of the maxillary bilateral first molars.

To replicate the impact of periodontal inflammation on early implant osseointegration, a peri-implant lesion model was created by applying various periodontal pathogenic bacteria around the implants, 10 days post-implantation. *Porphyromonas gingivalis* (ATCC 53977) and *F. nucleatum* (ATCC 25586) were cultivated within an anaerobic chamber. Adjusting the bacterial density, a logarithmic growth phase concentration of 3.3 × 10^9^*P. gingivalis*/ml was combined with an equivalent amount of *F. nucleatum*. These bacteria were allowed to interact for 5 min. Then, 200 μl of the bacterial solution was administered into the gingival sulcus situated between the implant and the gums once every 3 days with a total of six administrations. After the first time bacterial administration, the composite hydrogel was injected into the gingival sulcus. To ensure continuous presence in the gingival sulcus, the hydrogel was injected once every 3 days with a total of six treatments. Furthermore, NIR irradiation was applied on one Cu–Zn composite hydrogel group for 15 min with a power adjustment at a power adjustment at 0.30 W/cm^2^ to produce thermal stimulation at 45°C. All the animals were sacrificed on the 18th day and oral tissues were collected for further analysis.

The experiment was categorized into the following specific groups: (i) Blank group: Implants not infected by periodontal pathogens; (ii) Negative Control group: implants infected by periodontal pathogens and treated with Control hydrogel; (iii) Cu group: implants infected by periodontal pathogens and treated with Cu hydrogel; (iv) Zn group: implants infected by periodontal pathogens and treated with Zn hydrogel; (v) Cu–Zn group: implants infected by periodontal pathogens and treated with Cu–Zn hydrogel; (vi) Cu–Zn + Laser group: implants infected by periodontal pathogens and treated with Cu–Zn hydrogel with NIR laser irradiation.

### Micro-CT assessment

Upon completion of the 18-day implant placement period, the maxillary bones were harvested and immersed in a 10% formaldehyde solution for fixation. To evaluate peri-implant osseointegration, high-resolution micro-CT scans were performed using an animal micro-CT scanner (mCT-80, Scanco Medical). The scanning process utilized high-resolution settings, including a pixel matrix of 1024, a voxel size of 20 mm and a slice thickness of 20 mm. Three dimensional reconstruction techniques were employed for visualizing the peri-implant bone tissue. Micro-CT analysis facilitated the measurement of parameters including bone volume fraction (BV/TV%) and bone mineral density (BMD).

### Tissue sectioning and staining

The implants underwent decalcification using 10% EDTA solution. Subsequently, the implants were extracted and paraffin-embedded. Each specimen was sliced serially in the mesio-distal direction, with each slice having a thickness of 5 μm. A specific slice encompassing the region of the maxillary implant was chosen. Following overnight baking at 37°C, the slice was stored at 4°C. For observation under a light microscope, the slices underwent H&E staining, which allowed the assessment of inflammatory cell infiltration within the epithelial and connective tissues surrounding the implant. Additionally, Masson staining was applied to examine the organization and maturity of new bone tissue and collagen fibers around the implants. Furthermore, CD86 and CD206 were applied to examine the macrophages polarization.

### Statistical analysis

All experiments were conducted in triplicate, and the results are presented as mean ± standard deviation. Initially, Levene's test was employed to evaluate the homogeneity of variances. Subsequently, the comparison of statistical significance among groups was performed using one-way ANOVA followed by Bonferroni's *post hoc* test. A significance level of *P* < 0.05 was considered as statistically significant. The density of new blood vessels was analyzed using the AngioTool software, while GraphPad Prism 9.5.1 was utilized for generating statistical analyses, histograms, line graphs and violin plots.

## Results

### Characterization of cuprorivaite/hardystonite composite hydrogel (Cu–Zn)

**Figure 2.**
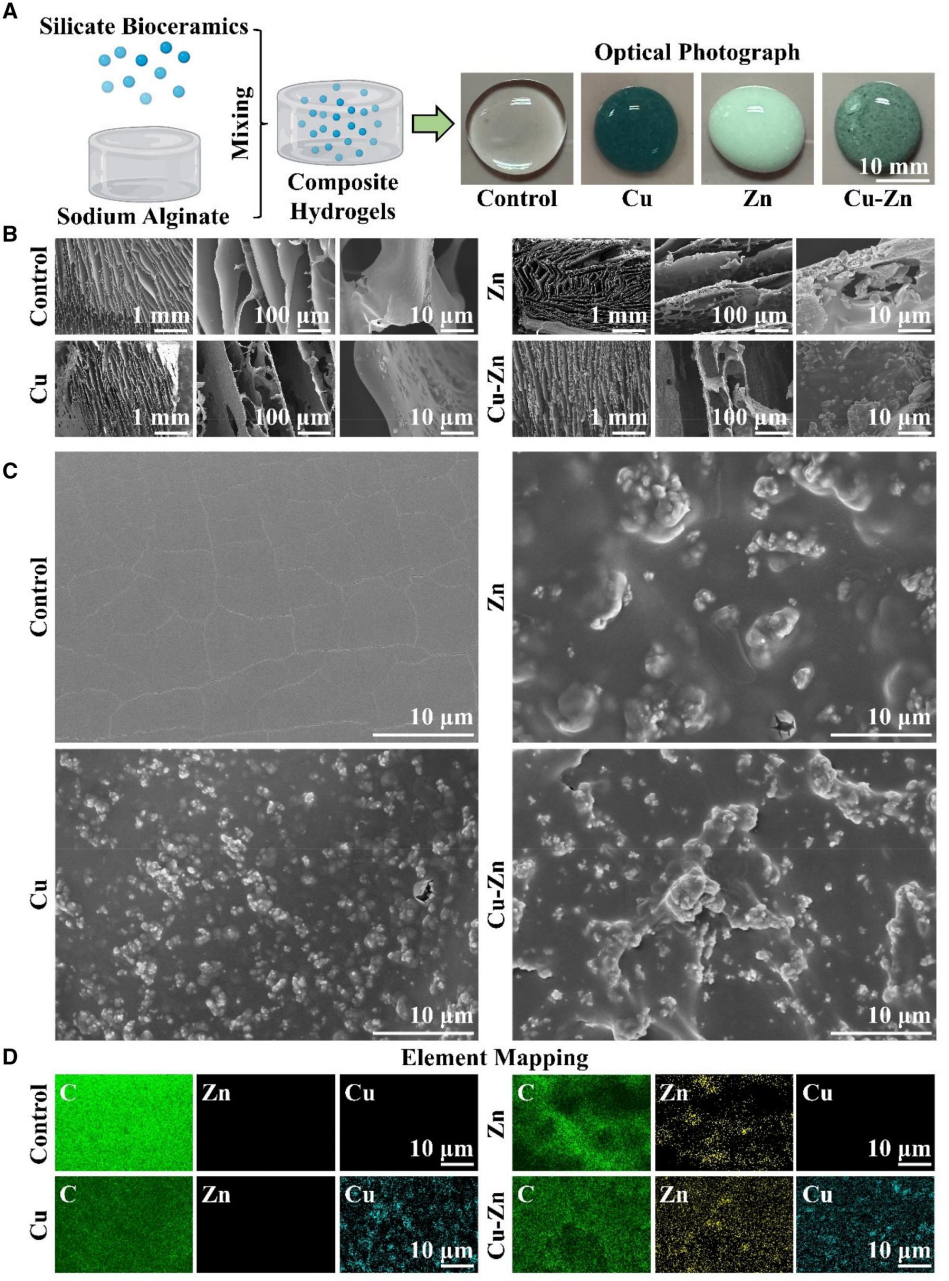
Preparation and characterization of cuprorivaite (CaCuSi_4_O_10_)/hardystonite (Ca_2_ZnSi_2_O_7_) composite hydrogel (Cu–Zn). Blank hydrogel (Control), cuprorivaite hydrogel alone (Cu) and hardystonite hydrogel alone (Zn) were utilized as Control groups for comparison. (**A**) Schematic diagram depicting the preparation process of the composite hydrogel and optical photograph of the composite hydrogel. (**B**) Scanning electron microscope (SEM) images of composite hydrogels. (**C**) The high-magnification SEM images of composite hydrogels. (**D**) The energy dispersive X-ray spectroscopy element distribution (C, Zn and Cu) of composite hydrogels in the high-magnification SEM area.

Cuprorivaite and hardystonite were successfully synthesized using the sol–gel method and the morphology and elemental distribution were characterized (Supplementary Figure S2). Subsequently, these bioceramics were homogeneously integrated into a sodium alginate hydrogel matrix. As shown in Figure 2A, the control sodium alginate hydrogel is transparent and colorless. In contrast, the cuprorivaite (Cu) containing hydrogel revealed a lake blue tint, while the hardystonite (Zn) composite hydrogel showed a white hue and the combination of cuprorivaite and hardystonite endowed the composite hydrogel (Cu–Zn) with distinct deep green color. Furthermore, the cuprorivaite—hardystonite combination significantly reduces the gelation time of the sodium alginate solution, with the gelation time decreasing from 13.61 ± 0.38 min for cuprorivaite (Cu) containing hydrogel (cross-linked with Zn^2+^ and Ca^2+^ ions) and 17.38 ± 1.26 min for hardystonite (Zn) composite hydrogel (cross-linked with Cu^2+^ and Ca^2+^ ions) to 10.02 ± 0.96 min for cuprorivaite/hardystonite (Cu–Zn) containing hydrogel (triple-ion cross-linking). In contrast, the control hydrogel without bioceramics relies on the external addition of CaCl_2_ to facilitate cross-linking, resulting in exceptionally rapid local cross-linking with a gelling time of 0.25 ± 0.07 min. Next, we compared the gelation time of the Cu–Zn composite hydrogel to the Cu hydrogel (containing 2% cuprorivaite) and the Zn hydrogel (containing 2% hardystonite) containing higher concentrations of bioceramics. The result revealed that the rate of gelation time in the Cu–Zn composite hydrogel still remained superior to that of both the Cu and Zn hydrogels (Supplementary Figure S3). These results suggest that Cu^2+^, Zn^2+^ and Ca^2+^ ions play a crucial role in enhancing the injectability of sodium alginate. From the time-dependent fluidity of the composite hydrogels, we found that the Cu, Zn and Cu–Zn composite hydrogels all lost fluid after approximately 16 min (Supplementary Figure S4). Before reaching this point of reduced fluidity, these composite hydrogels maintained good injectability (Supplementary Video S1). Furthermore, by correlating these results with gelation time of composite hydrogels, we concluded that the Cu, Zn and Cu–Zn composite hydrogel could maintain injectable within 13.61 ± 0.38, 17.38 ± 1.26 and 10.02 ± 0.96 min separately. In contrast, sodium alginate solution undergoes rapid gelation and loses fluidity within 1 min after addition of calcium chloride, which makes it difficult to be injected (Supplementary Figures S3 and S4; Supplementary Video S1). Furthermore, SEM examination indicates that all four hydrogels—Control, Cu, Zn and Cu–Zn—exhibit layered structures. Notably, the surfaces of the Cu, Zn and Cu–Zn hydrogels are rougher as compared to the Control group, with visible ceramic particles (Figure 2B). Additionally, while the bioceramic particles within the hydrogel appear uniform at macroscopic level, but not uniform in higher magnification. As a result, the element mapping image at high magnification reveals an uneven distribution of Cu and Zn elements within the hydrogel (Figure 2C). The Cu–Zn composite hydrogel possesses remarkable photothermal properties with a temperature rise up to 45°C under 808 nm NIR irradiation. Furthermore, the Cu composite hydrogel alone can reach 45°C under NIR irradiation. However, neither the Zn composite hydrogel nor the Control hydrogel exhibit any photothermal properties, suggesting that the photothermal property of the composite hydrogel is primarily attributed to the cuprorivaite (Supplementary Figure S5). Furthermore, we investigated the storage modulus (Gʹ) and loss modulus (Gʺ) of the hydrogels as a function of strain (Supplementary Figure S6A). The results showed that cuprorivaite or hardystonite did not significantly alter the rheological properties of the hydrogels. Similarly, the maximum stress of Cu–Zn composite hydrogel was 0.20 ± 0.05 N/mm^2^, which had no significant difference with other groups (Supplementary Figures S6B and S7). Remarkably, all composite hydrogels gradually release Cu^2+^ and Zn^2+^ ions with similar release rate (Supplementary Tables S2 and S3). The Cu–Zn composite releases more ${\text{SiO}}_{3}^{2-}$ in comparison to the individual Cu and Zn groups (Supplementary Table S4).

### Antibacterial property of Cu–Zn composite hydrogel

**Figure 3.**
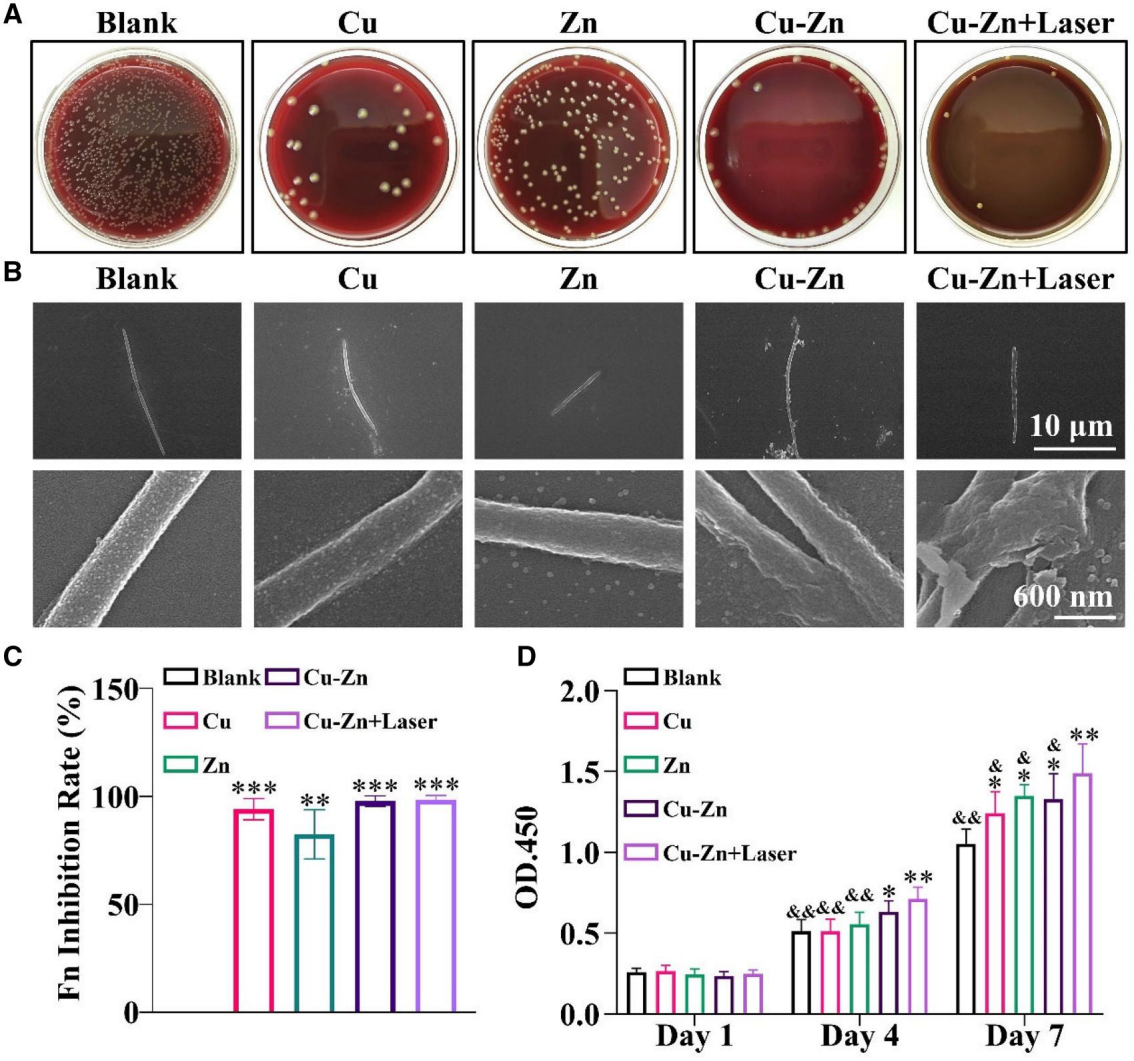
Antibacterial and biological activities of composite hydrogels. (**A**) Colony formation of the periodontal pathogen *F. nucleatum* co-cultured with composite hydrogels for 6 h. (**B**) Scanning electron microscopy (SEM) images of the morphological changes of *F. nucleatum* after treatment with composite hydrogels for 24 h. (**C**) Quantitative analysis of the inhibition rate of *F. nucleatum* by the composite hydrogel for 6 h. (**D**) Assessment of cell viability of rat bone marrow mesenchymal stem cells (rBMSCs) in the presence of composite hydrogels by CCK8 (*P < 0.05; **P < 0.01 compared with Blank group; & < 0.05, && < 0.01 compared with Cu–Zn + Laser group). The groups were as follows: (i) Blank: bacteria or cells without any material treatment; (ii) Cu: bacteria or cells treated with Cu composite hydrogel; (iii). Zn: bacteria or cells treated with Zn composite hydrogel; (iv) Cu–Zn: bacteria or cells treated with Cu–Zn composite hydrogel; (v) Cu–Zn + Laser: bacteria or cells treated with Cu–Zn composite hydrogel with NIR laser irradiation.

The antibacterial property of the composite hydrogel is critical for its application in early peri-implant lesion treatment. Hence, the antibacterial effectiveness of Cu–Zn composite hydrogels against *F. nucleatum*, a gram-negative, anaerobic oral bacterium, commensal to the human oral cavity, that plays a role in peri-implant lesion, was initially investigated. Figure 3A clearly demonstrates that the Cu–Zn composite hydrogel exhibits superior antibacterial activity, effectively eradicating a substantial portion of bacteria when cultured with composite hydrogels for 6 h as compared to the Control group. Notably, its antibacterial efficacy is further enhanced after NIR light irradiation (Figure 3A). Moreover, both Cu and Zn composite hydrogels display certain degrees of antibacterial activity, with the Cu composite hydrogel showing stronger efficacy as compared to the Zn composite hydrogel. SEM analysis reveals that, in comparison to the intact and smooth morphology of bacteria in the Blank group, bacteria in the Cu–Zn group undergo notable shrinkage and surface integrity disruption. NIR exposure additionally intensifies the antibacterial effect of the Cu–Zn group, and the Cu–Zn + Laser group shows nearly complete dissolution of bacteria. Furthermore, the sole Zn group exhibits a moderate capability to disrupt bacterial surfaces and most bacteria remain relatively intact, whereas the Cu group mildly compromises bacterial integrity locally, leading to surface-level bacterial dissolution (Figure 3B). These findings indicate that Cu^2+^ and Zn^2+^ play a key role for the antibacterial property of the composite hydrogels. It is only when these two components coexist that they can maximize cell destruction through a synergistic effect. Additionally, the combined thermal stimulation induced by NIR irradiation primarily contributes to the enhancement of the antibacterial properties of Cu^2+^ and Zn^2+^. Furthermore, quantitative analysis confirms that the Cu, Cu–Zn and Cu–Zn + Laser groups all demonstrate significantly higher antibacterial performance than the Zn group (Figure 3C).

### Activation of rBMSCs by Cu–Zn composite hydrogel in combination with photothermal heating

In addition to its remarkable antibacterial activity, the Cu–Zn composite hydrogel also retains noteworthy biological function to activate rBMSCs cells as revealed from the CCK-8 kit test outcomes. In comparison to the Blank group, the treatment of rBMSCs with the Cu–Zn group and Cu–Zn + Laser group leads to significant increase in cell viability, with a particularly pronounced effect observed in the Cu–Zn + Laser group. Notably, even on the 7th day, rBMSCs in the Cu–Zn + Laser group reveal exceptionally high cell viability (Figure 3D). Furthermore, a substantially higher cell counts in the Cu–Zn + Laser group was found as compared to other experimental groups according to the live-dead cell staining (Supplementary Figure S8A) and quantitative analysis further confirmed cell proliferation-promoting activity of Cu–Zn + Laser group (Supplementary Figure S8B). The observed proliferation of rBMSCs in the Cu–Zn + Laser group can be primarily attributed to mild thermal stimulation, which activates cellular ion channels [31, 32]. This activation may allow Zn^2+^, Cu^2+^ and ${\text{SiO}}_{3}^{2-}$ ions to interact more significantly with cells, ultimately promoting cell proliferation. This observation indicates that the Cu–Zn hydrogel can induce cell viability and promote cell proliferation when combined with NIR irradiation.

### Stimulation of osteogenic differentiation of rBMSCs by Cu–Zn composite hydrogel in combination with photothermal heating

**Figure 4.**
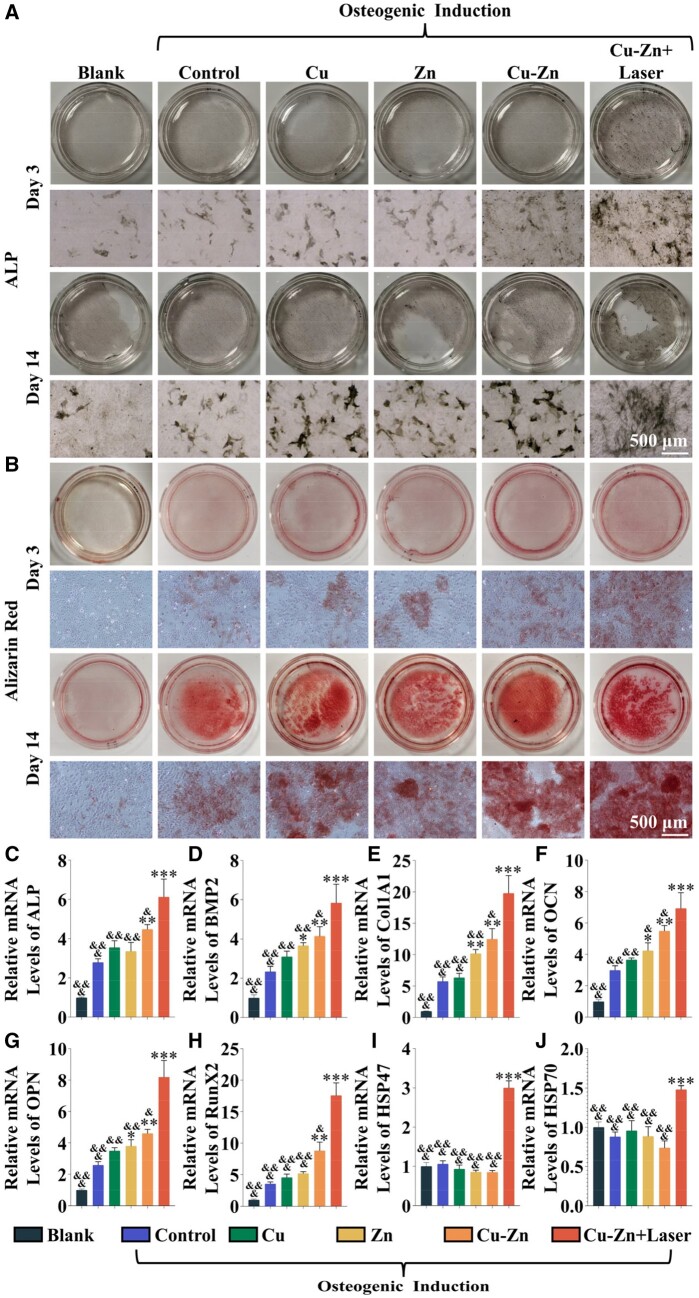
Evaluation of composite hydrogels for osteogenic differentiation. (**A**) Alkaline phosphatase staining of rBMSCs after 3 and 14 days of stimulation with composite hydrogels. (**B**) Alizarin red staining of rBMSCs after 3 and 14 days of stimulation with composite hydrogels. (**C**–**J**) The expression levels of osteogenesis-related genes (ALP, BMP2, Col1a1, OCN, OPN and RunX2) and heat shock proteins (HSP47 and HSP70) in rBMSCs stimulated with composite hydrogels for 14 days using RT-PCR (*P < 0.05; **P < 0.01; ***P < 0.001 compared with Control group; & < 0.05, && < 0.01 and &&& < 0.01 compared with Cu–Zn + Laser group). The groups were as follows: (i) Blank: cells without any treatment. Treated with osteogenic differentiation inducers: (ii) Control: cells treated with dexamethasone but without any material treatment; (iii) Cu: cells treated with dexamethasone and Cu composite hydrogel; (iv) Zn: cells treated with dexamethasone and Zn composite hydrogel; (v) Cu–Zn: cells treated with dexamethasone and Cu–Zn composite hydrogel; (vi) Cu–Zn + Laser: cells treated with dexamethasone and Cu–Zn composite hydrogel with NIR laser irradiation.

Materials with a significant capacity to enhance osteogenesis are crucial for accelerating implant osseointegration in the early treatment of peri-implant lesion. Therefore, composite hydrogels were co-cultured with rBMSCs, and the osteogenic differentiation potential was assessed through alkaline phosphatase staining and Alizarin Red S staining. In comparison to the other experimental groups, the Cu–Zn + Laser group demonstrated the highest activity to stimulate osteogenic differentiation, as evidenced by significantly enhanced ALP expression and calcification on both days 3 and 14. Furthermore, the Cu–Zn group emerges as the second most effective in fostering osteogenic differentiation, and the osteogenic differentiation capabilities of the Cu group and Zn group do not significantly differ from that of the fundamental osteogenic induction group (Control group) (Figure 4A and B). Subsequently, after a 14-day osteogenic induction, cellular RNA was extracted to assess the expression of osteogenesis-related genes using real-time PCR (Figure 4C–J). As compared to the basic osteogenic induction group (Control group), mRNA expressions of ALP, BMP2, Col1A1, OCN, OPN and Runx2 were significantly upregulated in the Cu–Zn group and Cu–Zn + Laser group, especially in the Cu–Zn + Laser group. In contrast, the expression of BMP2, Col1A1, OCN and OPN mRNA exhibited slight increases in the Zn group, while no significant differences were observed in the Cu group compared to the control group.

Furthermore, due to mild thermal stimulation from NIR irradiation, the Cu–Zn + Laser group exhibits notably increased expressions of heat shock proteins HSP47 and HSP70 mRNA. Similarly, western blot results align with this trend, confirming the protein expressions of COL1A1, OPN, RUNX2 and HSP47 (Supplementary Figure S9).

### Effect of Cu–Zn composite hydrogel with photothermal heating on peri-implant lesion *in vivo*

**Figure 5.**
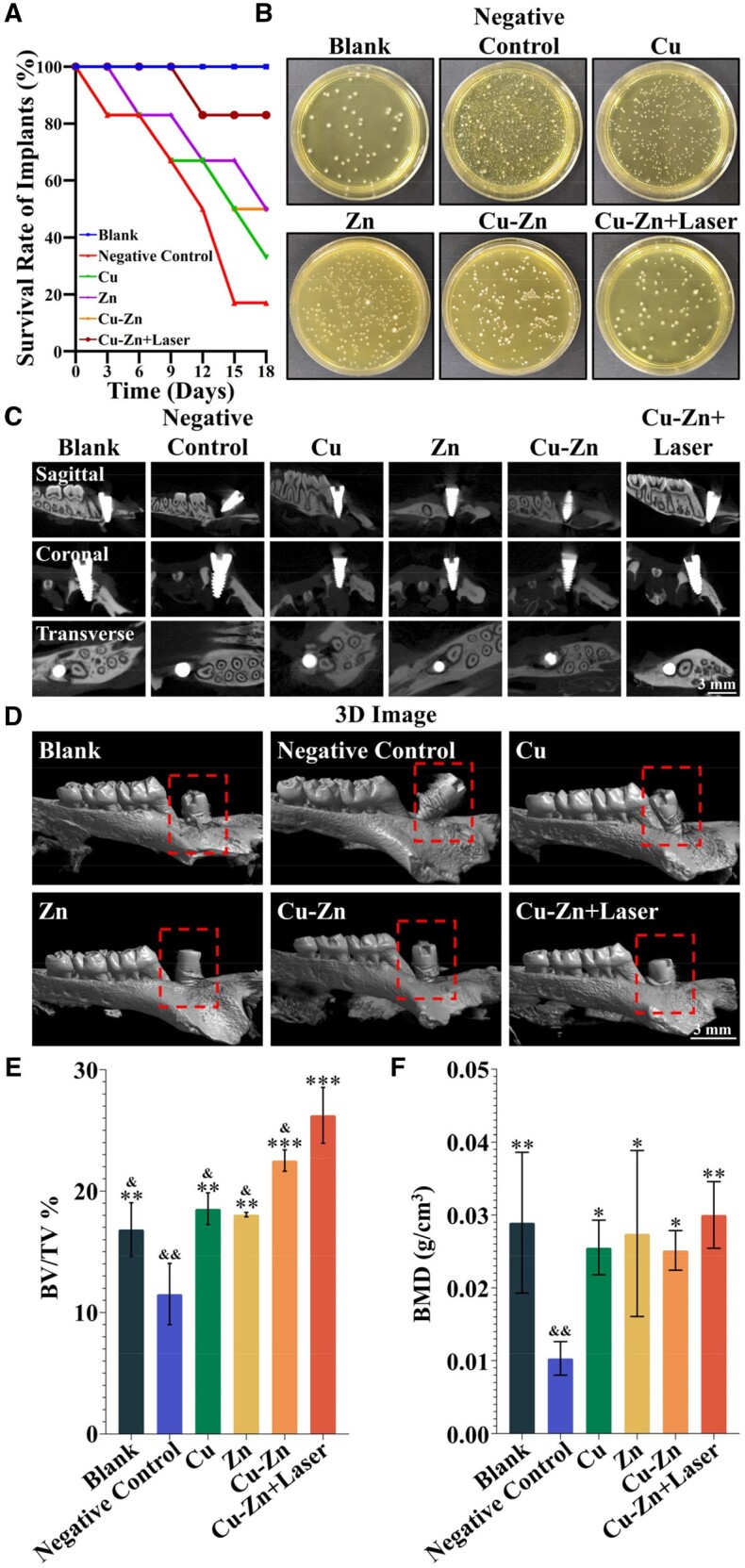
Implant survival rate, infection inhibition and osseointegration of the implant after treatment with composite hydrogels. (**A**) Comparison of the survival rate of implants between different groups after 18 days (*n* = 12). (**B**) The bacterial content around the implants at Day 18. (**C**) Micro-CT images illustrate the osseointegration of maxillary implants in each group from different sections. (**D**) Three-dimensional reconstructions demonstrate the bone condition around the implant and the exposure of the implant thread in each group. (**E**) Quantitative analysis of bone volume/tissue volume (BV/TV) around the implants in each group. (**F**) Quantitative analysis of bone mineral density (BMD) around the implants in each group (*P < 0.05; **P < 0.01; ***P < 0.001 compared with Negative Control group; & < 0.05, && < 0.01 compared with Cu–Zn + Laser group). The groups were as follows: (i) Blank group: implants not infected by periodontal pathogens; (ii) Negative control group: implants infected by periodontal pathogens and treated with control hydrogel; (iii) Cu group: implants infected by periodontal pathogens and treated with Cu hydrogel; (iv) Zn group: implants infected by periodontal pathogens and treated with Zn hydrogel; (v) Cu–Zn group: implants infected by periodontal pathogens and treated with Cu–Zn hydrogel; (vi) Cu–Zn + Laser group: implants infected by periodontal pathogens and treated with Cu–Zn hydrogel with NIR laser irradiation.

In order to confirm the therapeutic effect of the Cu–Zn composite hydrogel in treating the peri-implant lesion, we used a SD rat early peri-implant lesion model by introducing periodontal pathogenic bacteria as shown in Supplementary Figure S1. Implant survival rates were determined on Day 18 after animal sacrifice with 12 implants in each group. Notably, the Blank group, free from additional exposure to periodontal pathogenic bacteria, maintained implant integrity during the experiment period. Conversely, in the Negative Control group afflicted by peri-implant lesion, only 2 out of the 12 implants remained intact, resulting in a survival rate of 16.67%. In contrast, all the experimental groups revealed increased survival rates, and the Cu, Zn, Cu–Zn and Cu–Zn + Laser groups revealed implant survival rates of 33.33%, 50%, 50% and 83.33%, respectively. The Cu–Zn + Laser group exhibited the highest implant survival rate indicating its superior therapeutic efficacy (Figure 5A).

### Inhibition of infection *in vivo* by Cu–Zn composite hydrogel with photothermal heating

The antibacterial activity of the Cu–Zn composite hydrogel appears to be a key factor in its effectiveness against peri-implant lesion. To assess its performance in the body, we gathered plaque from around the implants just before the rats were euthanized on the 18th day. The results revealed that the Cu–Zn + Laser group had notably fewer bacteria than the negative control group, almost as few as the Blank group. Conversely, the Cu, Zn and Cu–Zn groups showed only a modest reduction in bacterial levels as compared to the negative control group, and the Cu–Zn group displayed a slight increase in its antibacterial effect as compared to Cu and Zn groups (Figure 5B and Supplementary Figure S10).

### Stimulation of osseointegration *in vivo* by Cu–Zn composite hydrogel with photothermal heating

Micro-CT analysis was employed to evaluate implant osseointegration at Day 18. In the Cu–Zn + Laser group, significantly more new bone formation was observed with increased bone density of the surrounding AB. In contrast, the Negative Control group exhibited a low-density image along the entire length of the implant, devoid of any new bone formation. Moreover, within the Cu, Zn and Cu–Zn groups, limited new bone formation occurred around the root and neck of the implant, characterized by low bone density (Bone is depicted in white, with increased whiteness indicating higher bone density.) (Figure 5C and Supplementary Figure S11). Three-dimensional reconstruction further indicated implant dislodgement from the alveolar socket, with complete thread exposure in the Negative Control group. In contrast, the Cu–Zn + Laser group revealed nearly identical Implant condition as that of the peri-implant lesion-free Blank group with no thread exposure around the implant. The Cu, Zn and Cu–Zn groups showed improved implant condition as compared to the Negative Control group with 3–5 rings of exposed threads around the implant neck (Figure 5D). Bone volume fraction (BV/TV%) revealed a significant enhancement in all experimental groups as compared to the Negative Control group, suggesting a prevalence of bone anabolic metabolism over catabolism, leading to an overall increase in bone mass (Figure 5E). The Cu–Zn + Laser group exhibited the most substantial elevation among these groups. In terms of BMD, all experimental groups displayed a remarkable increase as compared to the Negative Control group (Figure 5F).

**Figure 6.**
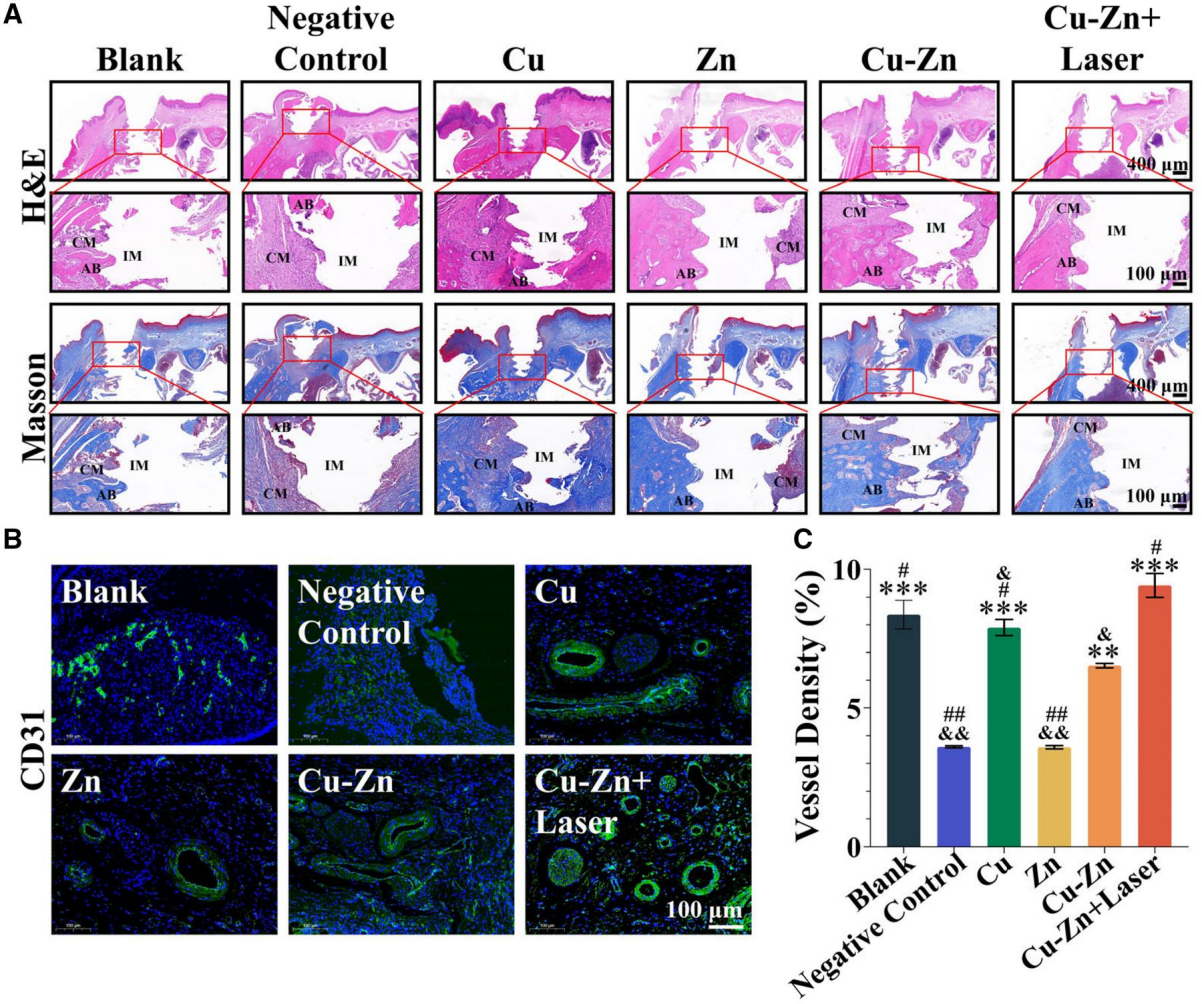
Effect of composite hydrogel on peri-implant bone tissue integration and angiogenesis after 18 days. (**A**) Representative images of H&E staining and Masson staining illustrating tissue integration around implant. (**B**) Representative immunofluorescence staining images showing angiogenesis around implants. (**C**) Quantitative analysis of neovascular density according to CD31 staining (**P < 0.01; ***P < 0.001 compared with negative control group; & < 0.05, && < 0.01 compared with Cu–Zn + Laser group; # < 0.05, ## < 0.01 compared with Cu–Zn group) (AB: alveolar bone; CM: collagen matrix; IM: implant). The groups were as follows: (i) Blank group: implants not infected by periodontal pathogens; (ii) Negative control group: implants infected by periodontal pathogens and treated with Control hydrogel; (iii) Cu group: implants infected by periodontal pathogens and treated with Cu hydrogel; (iv) Zn group: implants infected by periodontal pathogens and treated with Zn hydrogel; (v) Cu–Zn group: implants infected by periodontal pathogens and treated with Cu–Zn hydrogel; (vi) Cu–Zn + Laser group: implants infected by periodontal pathogens and treated with Cu–Zn hydrogel with NIR laser irradiation.

In addition, implant osseointegration was evaluated using H&E staining and Masson staining. Within the Blank, Cu, Zn, Cu–Zn and Cu–Zn + Laser groups, a well-defined thread structure, indicative of successful integration between periodontal tissue and the implant, was consistently observed. Interestingly, the thread structure within the Cu–Zn group and Cu–Zn + Laser group was predominantly comprised of AB tissue, signifying near-complete osseointegration of the implant. More interestingly, the Cu–Zn + Laser group exhibited a significantly greater new bone volume as compared to the Cu–Zn group, highlighting the substantial improvement of therapeutic efficacy attributed to the mild thermal effect induced by NIR. Additionally, a limited amount of new AB can be discerned around the threads in both the Cu and Zn groups, while in Negative Control groups primarily only collagen-based matrix (CM) was observed. Noteworthy, the Negative Control group displayed no thread structure, which was a factor largely contributing to the complete implant failure (Figure 6A).

### Angiogenesis effect of Cu–Zn composite hydrogel with photothermal heating *in vivo*

Angiogenesis plays a pivotal role in ensuring sufficient nourishment of the bone tissue surrounding the implant. Therefore, the capacity of the composite hydrogel to stimulate angiogenesis is of great importance. Remarkably, our results demonstrated that the Cu–Zn + Laser group exhibited the most pronounced CD31-positive staining on Day 18 as compared to all other groups (Figure 6B). Moreover, quantitative analysis of CD31 staining indicated a significantly higher number of blood vessels in the Cu–Zn + Laser group compared to the Cu–Zn group without laser irradiation, underscoring the substantial enhancement in therapeutic efficacy attributed to the thermal effect induced by NIR (Figure 6C). Furthermore, in comparison to the Negative Control group, the Zn group alone showed no stimulating effect on angiogenesis, while the Cu group alone significantly enhanced blood vessel formation, suggesting a dominant role of the Cu group in promoting angiogenesis. It is noteworthy that the blood vessel density of the Cu group was higher than that of the Cu–Zn group but lower than that of the Cu–Zn + Laser group. The blood vessel density in the Cu group is higher than that in the Cu–Zn group due to the fact that Zn^2+^ may act as an antagonist to Cu^2+^ in stimulating angiogenesis [33]. Furthermore, the blood vessel density in the Cu–Zn + Laser group is superior to the Cu–Zn group, owing to the mild thermal stimulation produced by NIR, which could activate angiogenesis factors including vascular endothelial growth factor (VEGF) and endothelial nitric oxide synthase (eNOS) in endothelial cells [34]. This result suggests that, while the combination of Cu^2+^ and Zn^2+^ ions may not exhibit a positive synergistic effect in promoting angiogenesis, the photothermal heating significantly enhanced angiogenic activity of the triple-ion combination of Cu^2+^, silicate and Zn^2+^ ions, although Zn^2+^ ions may not contribute to the enhancement of angiogenesis without heating (Figure 6C).

### Inhibition of inflammatory response *in vivo* by Cu–Zn composite hydrogel with photothermal heating

**Figure 7.**
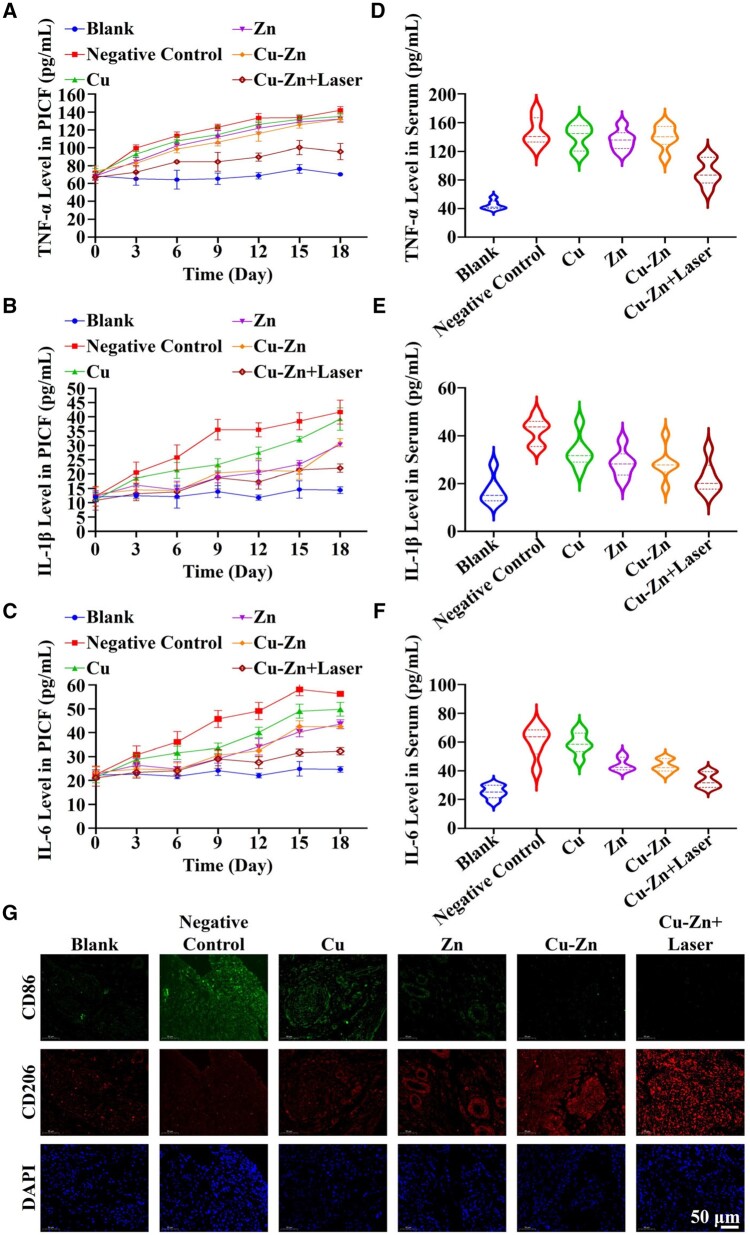
The expression of inflammatory factors in the gingival crevicular fluid and blood, and the immunofluorescence staining of macrophages in the soft tissue surrounding the implant after treatment with composite hydrogels. (**A**–**C**) Enzyme-linked immunosorbent assay (ELISA) results showing the levels of inflammatory factors (TNF-α, IL-1β and IL-6) from Day 0 to Day 18 in the peri-implant gingival crevicular fluid (PICF) after infection with periodontal pathogenic bacteria following implant placement (*n* = 12). (**D**–**F**) ELISA results showing the levels of inflammatory factors (TNF-α, IL-1β and IL-6) at Day 18 in the serum of rats after implant placement (*n* = 6). (**G**) Representative CD86 or CD206 positive immunofluorescence staining images of the tissues around implants at Day 18. The groups were as follows: (i) Blank group: implants not infected by periodontal pathogens; (ii) Negative control group: implants infected by periodontal pathogens and treated with control hydrogel; (iii) Cu group: implants infected by periodontal pathogens and treated with Cu hydrogel; (iv) Zn group: implants infected by periodontal pathogens and treated with Zn hydrogel; (v) Cu–Zn group: implants infected by periodontal pathogens and treated with Cu–Zn hydrogel; (vi) Cu–Zn + Laser group: implants infected by periodontal pathogens and treated with Cu–Zn hydrogel with NIR laser irradiation.

The anti-inflammatory effect of the composite hydrogel is closely linked to its therapeutic effect on peri-implant lesion. Remarkably, the results showed that the level of inflammatory factors (TNF-α, IL-1β and IL-6) in the gingival crevicular fluid from Day 0 to Day 18 in Cu–Zn + Laser group consistently maintained lower than other groups, and close to the Blank group. In contrast, the other groups experienced a gradual increase of inflammatory factors in the gingival crevicular fluid over time, although the Cu group, Zn group and Cu–Zn group all demonstrated a modest anti-inflammatory effect as compared to the Negative Control group (Figure 7A–C). Additionally, the Cu–Zn + Laser group also exhibited a pronounced anti-inflammatory effect in the serum of rats on Day 18, and the level of TNF-α, IL-1β and IL-6 are clearly lower than in other groups (Figure 7D–F). Furthermore, the impact of the composite hydrogel on macrophage polarization was assessed through CD86/CD206 double staining. It was revealed that the Cu–Zn group and Cu–Zn + Laser group displayed a substantial reduction in CD86-positive staining as compared to the other groups, accompanied by a notable increase in CD206-positive staining (Figure 7G). Given that CD206 serves as the marker of M2 macrophages and CD86 as the marker of M1 macrophages, the results suggest that the thermionic effect may also inhibit inflammation by significantly inhibiting M1 polarization of macrophages while promoting M2 polarization of macrophages.

## Discussion

Peri-implant lesion is an inflammatory condition caused by microbial plaque that disrupts the integration of soft and hard tissues around the implant, like the pathogenesis of periodontitis. As a result, clinicians often treat peri-implant lesion using periodontitis therapies. However, despite their similar pathogenesis, peri-implant lesion poses a heightened risk of bone loss, culminating in the failure of implant osseointegration. For this reason, treatment programs tailored for periodontitis, which emphasize the precise delivery of antibiotics and other antibacterial agents, cannot be directly applied to the peri-implant lesion [35–37]. To address these challenges, our study introduces an injectable dual-bioceramic (Cuprorivaite/Hardystonite) composite hydrogel (Cu–Zn). This hydrogel showed unique thermionic effect due to the photothermal function of the bioceramics under NIR radiation and the release of bioactive Cu^2+^, Zn^2+^ and ${\text{SiO}}_{3}^{2-}$ ions from the composites owing to the degradation properties of the bioceramics. This effect is remarkably effective in eradicating bacteria, reducing inflammation, and promoting osteogenic differentiation, providing significant therapeutic benefits for the treatment of peri-implant lesion.

This bioactive composite hydrogel is formed by utilizing the cross-linking between divalent metal ions released from the two bioceramics and alginate molecules. The traditional way to prepare alginate hydrogel is mixing calcium chloride and alginate, and due to the quick reaction of calcium ions with alginate, the hydrogel is not injectable as observed in our experiments. In our previous studies we have found that although the mixing of some bioceramics with alginate may result in cross-linking reaction between metal ions released from bioceramics and alginate, the reaction is too slow and the gelling time is too long so that the injectability is very poor, and an assistant agent such as D-gluconic acid d-lactone or acidic amino acids is required to accelerate the gelling process in order to obtain an injectable hydrogel [38, 39]. It is interesting to see in the present study that our composite hydrogel system formed dual-bioceramic/alginate composite hydrogel with good injectability without adding any assistant agent. This is probably due to the suitable ion release rate of the cuprorivaite/hardystonite ceramic combination, in which one releases Ca^2+^ and Cu^2+^ and the other release Ca^2+^ and Zn^2+^ resulting in a triple-ion mixture for gradual cross-linking of alginate with a suitable rate. With just a 2% loading of cuprorivaite/hardystonite bioceramics combination, we achieved effective cross-linking in approximately 10 min, an ideal duration for injectable hydrogels, preventing post-implantation outflow and preserving treatment efficacy. In contrast, single-bioceramic cross-linked hydrogels containing 2% bioceramic loading need over 10-min to form gels. This phenomenon may be attributed to the inherent solubility characteristics of these two bioceramics. When considering hydrogels of the same volume and a total 2% ceramic addition, the Cu–Zn composite hydrogel has a concentration of 1% for each ceramic, whereas the Cu or Zn composite hydrogels have a ceramic concentration of 2%. Taking into account the low solubility of these ceramics, the combined ion concentration of Cu and Zn may be higher than the Cu ion concentration in the Cu composite hydrogel and the Zn ion concentration in the Zn composite hydrogel. Furthermore, it is noteworthy that all the composite hydrogels possess a layered structure. The formation of the layered structure is related to the concentration of the sodium alginate solution, which is known to form layered structure by spontaneous assembly when the concentration of sodium alginate exceeds 10 mg/ml [40]. Our results further confirmed that the addition of bioceramics did not alter the structure of the alginate hydrogel.

When addressing early-stage peri-implant inflammation, the prevention of bone resorption around the implant and facilitation of osseointegration are critical for the final success of the implant. The combinations of antibiotics with growth factors have been used to eliminate plaque from the implant surface and restore missing bone tissue around the implant [6, 9]. However, this treatment approach has significant limitations. Firstly, drug resistance is a big concern associated with the application of antibiotics [41–48]. Secondly, the administration of large doses of growth factors that stimulate bone regeneration (such as BMP-2 and TGF-β) can induce inflammatory reactions *in vivo* [49, 50]. Therefore, the combination of antibiotics and growth factors for peri-implant lesion treatment often results in a lengthy treatment cycle and is less effective in preventing bone resorption. In contrast, the primary strength of the Cu–Zn composite hydrogel developed in this study lies in its remarkable capacity to simultaneously inhibit bacteria and enhance osseointegration though thermionic effect. Mechanistically, the release of low-dose Zn^2+^ and Cu^2+^ and ${\text{SiO}}_{3}^{2-}$ from the Cu–Zn composite hydrogel plays a dual role in promoting osseointegration and inhibiting bacteria growth. Notably, Zn^2+^ primarily enhances osteogenic differentiation of mesenchymal stem cells, while Cu^2+^ promotes angiogenesis to support bone regeneration. ${\text{SiO}}_{3}^{2-}$ ions act as enhancers, synergistically improving the functionalities of Zn^2+^ and Cu^2+^ ions. More importantly, the photothermal property of the composite hydrogel allows creation of thermionic effect by NIR irradiation, which significantly enhances the bioactive effects of Zn^2+^ and Cu^2+^ and ${\text{SiO}}_{3}^{2-}$, including antibacterial properties, osteogenic differentiation and angiogenesis. The enhancement of ion bioactivity by NIR irradiation may be attributed to the fact that mild thermal stimulation at 45°C significantly increases bacterial biofilm permeability [51, 52] and activates cellular cation and anion channels, such as transient receptor potential vanilloid receptor 4 (TRPV4) and anoctamin 1 (ANO1) [31, 32], so that Zn^2+^, Cu^2+^ and ${\text{SiO}}_{3}^{2-}$ ions can more significantly interact with bacteria or cells. Our previous investigations have shown that Cu^2+^ and ${\text{SiO}}_{3}^{2-}$ ions enhance wound healing of infectious wounds through the inhibition of *Staphylococcus aureus* and *Escherichia coli* as well as stimulation of angiogenesis [18]. In this study, we demonstrated that triple-ion thermionic effect is stronger than single-ion thermionic effect and are able to effectively inhibit oral bacteria and enhance osteointegration of dental implants. This result suggests that biomaterials designed using the thermionic effect could be suitable for a variety of tissue regeneration environments. Furthermore, our study has also verified that the thermal effect with multiple ions can generate a stronger and more extensive synergistic effect than single ions.

Given that complete eradication of periodontal pathogenic bacteria in the oral cavity is often unattainable, local or systemic antibiotic treatment remains an indispensable step in peri-implant lesion management. However, clinical data reveal that even with antibacterial intervention, nearly 40% of peri-implant lesion cases exhibit persistent and intense inflammatory responses leading to substantial bone loss [53]. This phenomenon can be attributed to the common occurrence in bacterial-mediated diseases where inflammation persists for a certain period post-bacterial clearance [54]. Consequently, an effective peri-implant lesion treatment should not only inhibit bacteria growth, but also suppress inflammation. In our previous study, we observed that Cu^2+^ ions have activity to stimulate inflammatory response and enhance bacteria elimination by inducing M1 polarization of macrophages, while Zn^2+^ ions have activity to suppress inflammation by inducing M2 polarization of macrophages [28]. Our present study demonstrates that the combination of Cu^2+^, Zn^2+^ and ${\text{SiO}}_{3}^{2-}$ ions simultaneously released from the cuprorivaite and hardystonite bioceramics within Cu–Zn composite hydrogel significantly enhance macrophage M2 polarization under NIR stimulation. This result indicates that the thermionic effect has unique potential in regulating immune responses in favor of osteointegration.

## Conclusion

In this study, a novel dual-bioceramics/alginate composite hydrogel was designed, which contains cuprorivaite and hardystonite bioceramics and is injectable due to triple-ion cross-linking. This bioactive composite hydrogel exhibits exceptional photothermal property and the capability to release bioactive ions (Cu^2+^ and Zn^2+^ and ${\text{SiO}}_{3}^{2-}$), which generated unique thermionic effect. When the composite hydrogel was used for the treatment of peri-implant lesions, the thermionic effect effectively eliminates periodontal pathogenic bacteria and inhibits inflammation, while simultaneously enhances peri-implant osseointegration as compared to materials treatment without mild photothermal heating, thereby significantly increases the survival rate of implants. Considering different biological activities of three different ions, Cu^2+^ and Zn^2+^ ions mainly attributed to antibacterial effect, while Zn^2+^ ions mainly contributed to the role of suppressing inflammation, and Cu^2+^ and ${\text{SiO}}_{3}^{2-}$ ions are more bioactive in stimulating angiogenesis. More importantly, the mild heating significantly enhances the function of each type of ions and in particular the ion combination, which indicates that the design of biomaterials with thermionic function is an effective approach for treatment of peri-implant lesion for increased success rate of dental implants, and this thermionic effect-based approach might also be applicable for the repair of other bacteria infected tissues.

## Supplementary Material

rbae028_Supplementary_Data
