# Supplementary material for: Cuprorivaite/hardystonite/alginate composite hydrogel with thermionic effect for the treatment of peri-implant lesion
Source: Regen Biomater. 2024 Mar 21;11:rbae028. doi: 10.1093/rb/rbae028 (PMC11007117; doi:10.1093/rb/rbae028)
Supplement: rbae028_Supplementary_Data [file rbae028_supplementary_data.zip › Supporting Information 2024-3-13.docx]

**Supporting Information**

**Cuprorivaite/Hardystonite/Alginate Composite Hydrogel with Thermionic Effect for the Treatment of Peri-implant** **Lesion**

*Yiru Xia^1,2,3,^*^ξ^*, Zhaowenbin Zhang^6,7,8,^*^ξ^*, Kecong Zhou^1, 2, 3^, Zhikai Lin^1, 2, 3^, Rong Shu^1, 2, 3^, Yuze Xu^6,7,8^, Zhen Zeng^6,7,8,9^, Jiang Chang ^6, 7, 8,^** *and Yufeng Xie^1,2,3,4,5^* *

1. Department of Periodontology, Shanghai Ninth People's Hospital, Shanghai Jiao Tong University School of Medicine, Shanghai, China.
2. National Clinical Research Center for Oral Diseases, Shanghai Key Laboratory of Stomatology & Shanghai Research Institute of Stomatology, Shanghai, China.
3. Shanghai Engineering Research Center of Advanced Dental Technology and Materials. Shanghai, China
4. Department of Periodontology, Shanghai Stomatological Hospital & School of Stomatology, Fudan University
5. Shanghai Key Laboratory of Craniomaxillofacial Development and Diseases, Fudan University
6. Joint Centre of Translational Medicine, the First Affiliated Hospital of Wenzhou Medical University, Wenzhou, 325000, China.
7. Zhejiang Engineering Research Center for Tissue Repair Materials, Wenzhou Institute, University of Chinese Academy of Sciences, Wenzhou, 325000, China.
8. State Key Laboratory of High-Performance Ceramics and Superfine Microstructure, Shanghai Institute of Ceramics, Chinese Academy of Sciences, Shanghai 200050, People’s Republic of China.
9. Key Laboratory of Rehabilitation Medicine in Sichuan Province, West China Hospital, Sichuan University, Chengdu, People’s Republic of China.

ξ These authors have contributed equally to this work and share the first authorship.
* Corresponding Author: [jchang@mail.sic.ac.cn](mailto:jchang@mail.sic.ac.cn); [yufengxie_123@163.com](mailto:yufengxie_123@163.com).

**
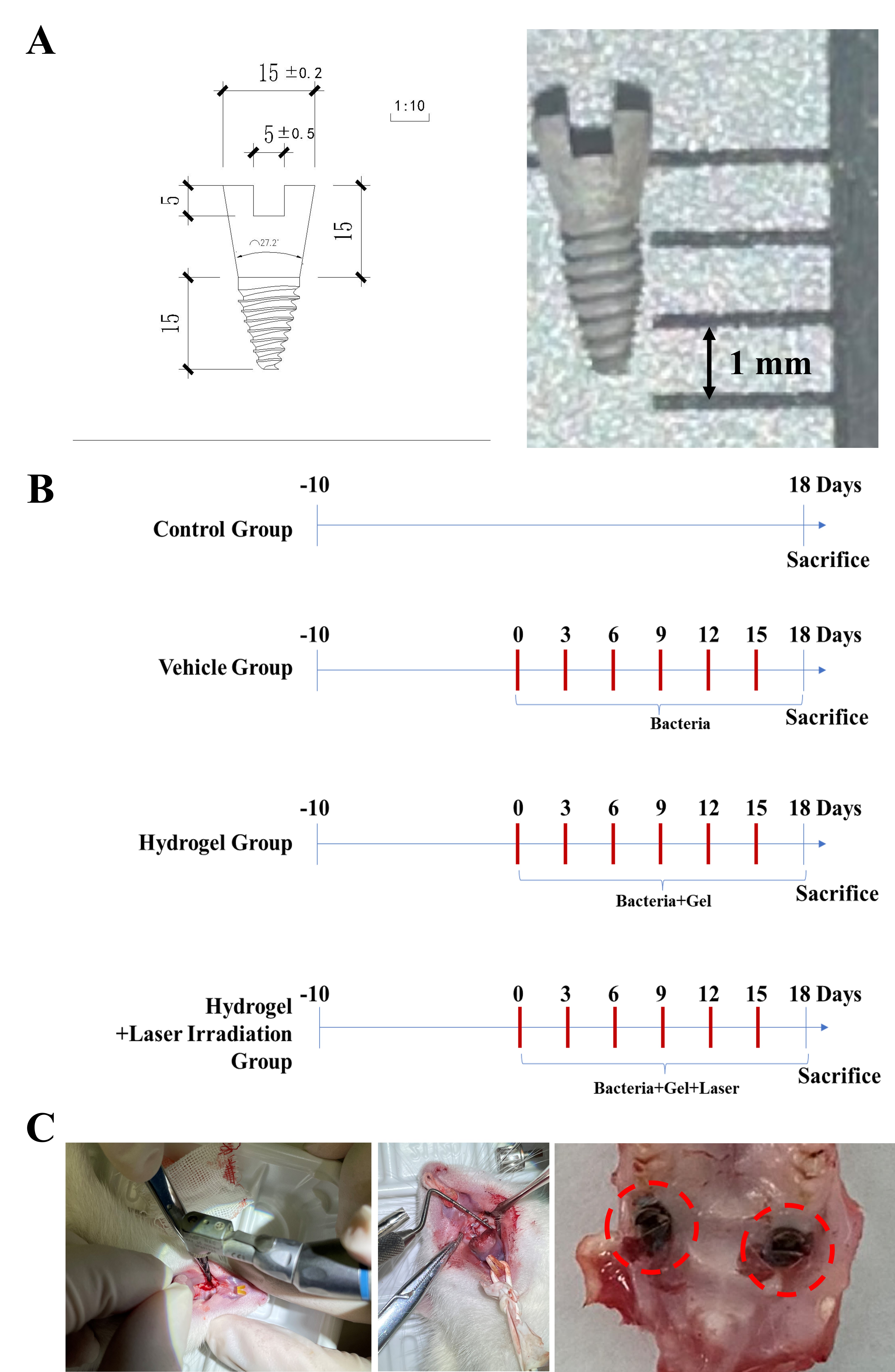
**

**Figure S1.** (A) Schematic and optical photographs of the implant. (B) The protocol for animal experiments. (C) The process of the animal experiment.

**
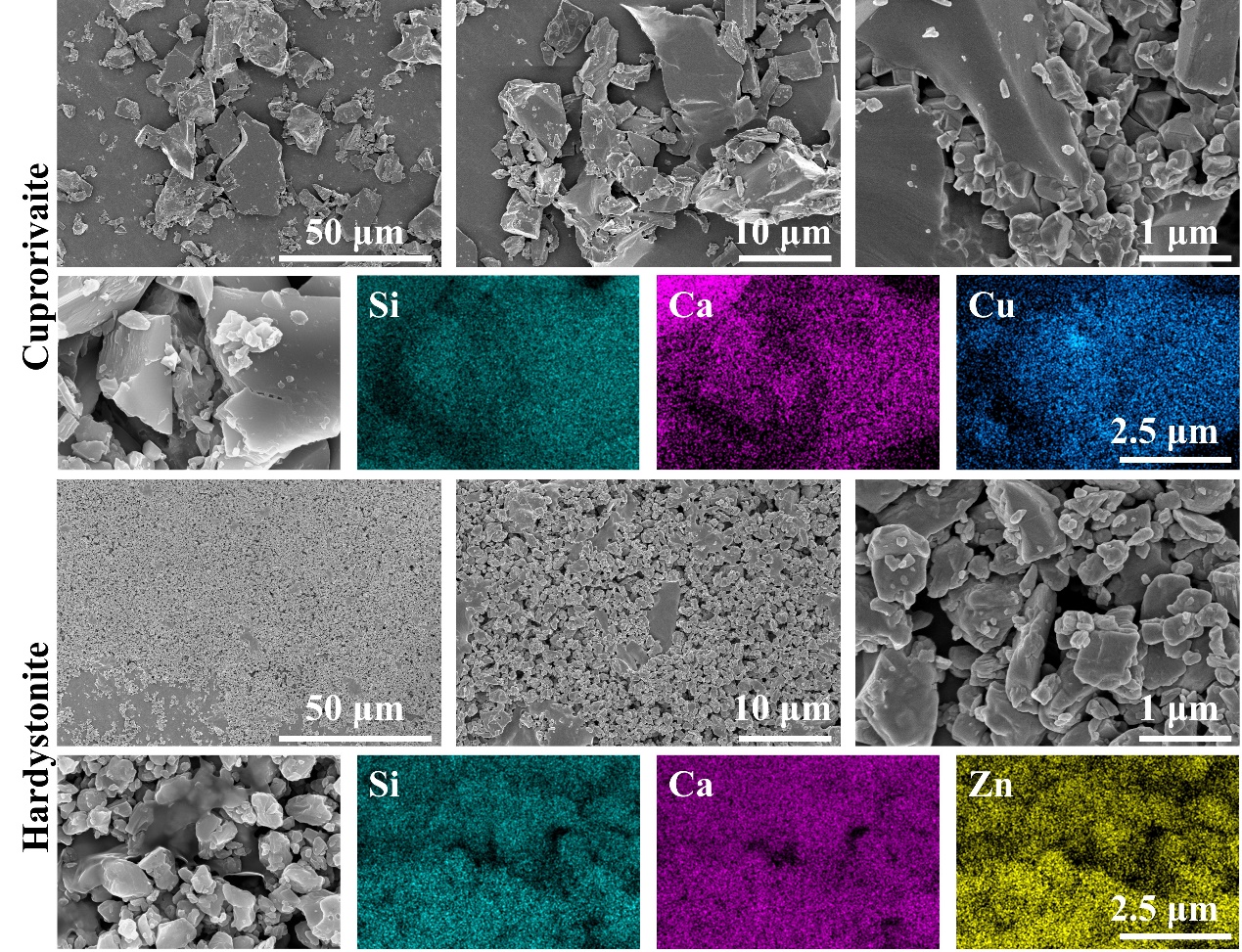
**

**Figure S2.** Scanning electron microscope (SEM) images of the cuprorivaite (CaCuSi_4_O_10_) and hardystonite (Ca_2_ZnSi_2_O_7_) and elemental distribution analysis of the cuprorivaite (CaCuSi_4_O_10_) and hardystonite (Ca_2_ZnSi_2_O_7_) using Energy Dispersive Spectrometer (EDS) mapping.


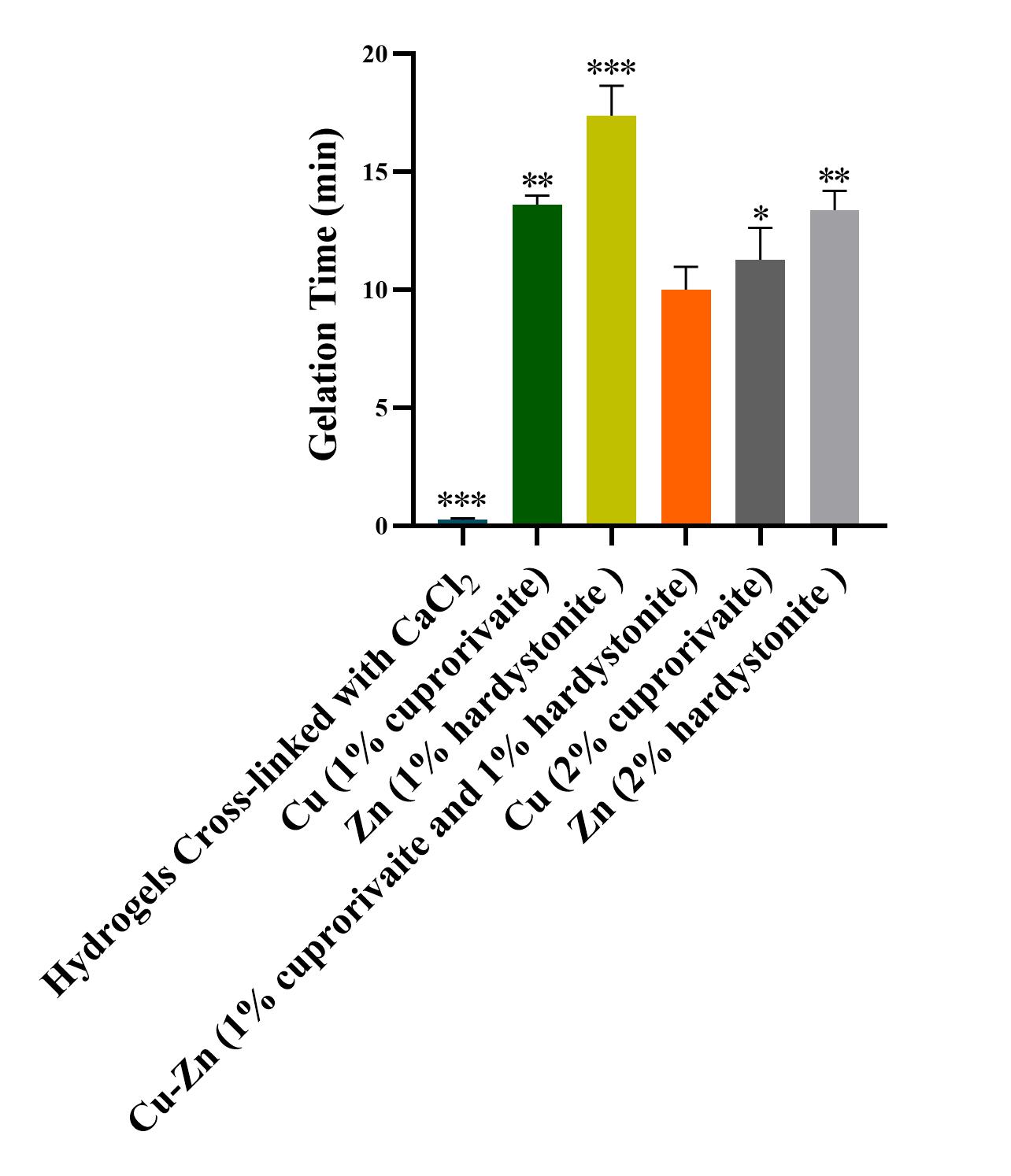


**Figure S3.** Gelation time of composite hydrogels. *p < 0.05 ;**p < 0.01 ;***p < 0.001 compared with Cu-Zn group.

1. Cu: cuprorivaite hydrogel;

2. Zn: hardystonite hydrogel;

3. Cu-Zn: cuprorivaite/hardystonite composite hydrogel.


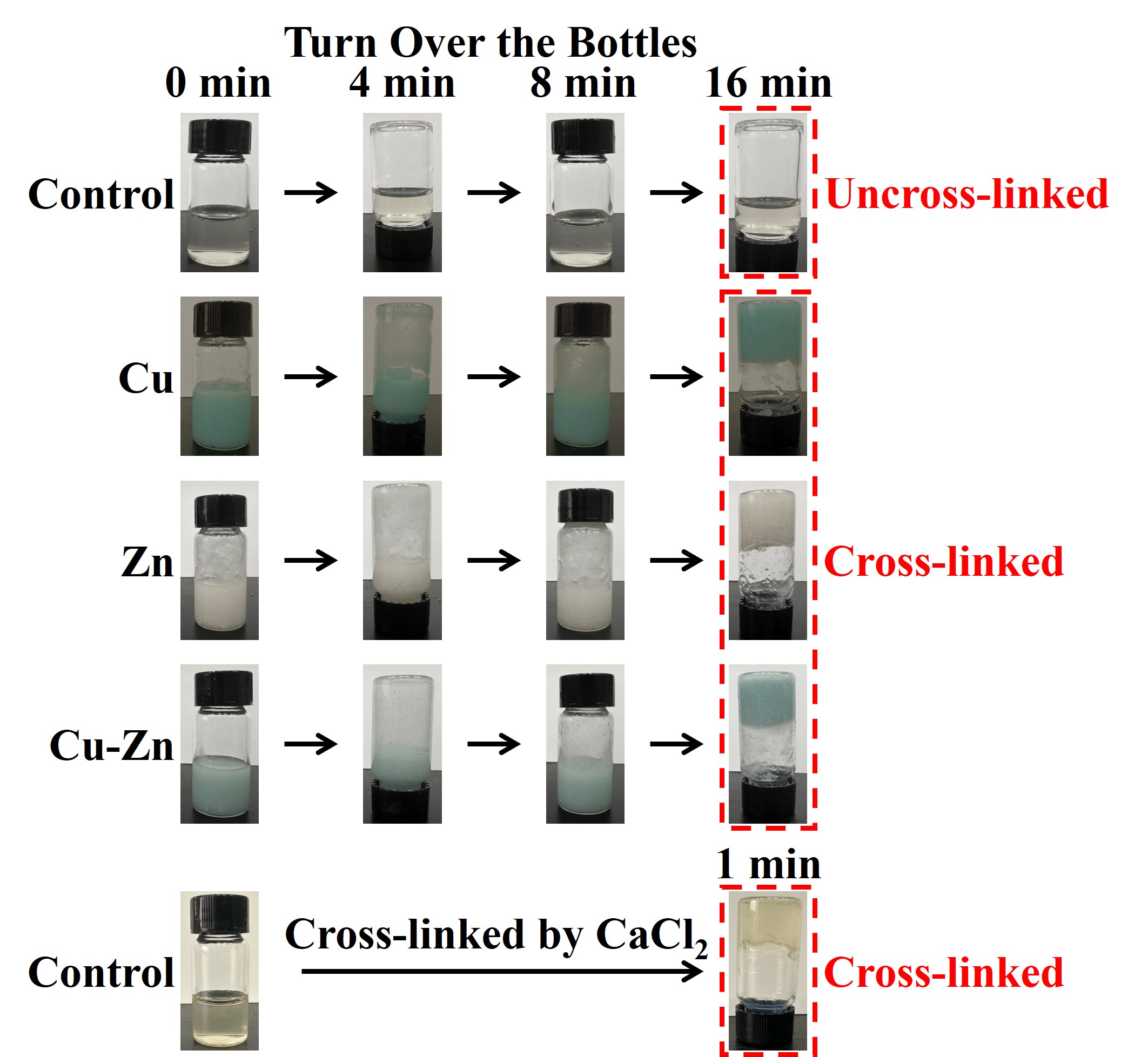


**Figure S4.** Time-dependent fluidity of composite hydrogels.

1. Control: sodium alginate solution;

2. Cu: cuprorivaite hydrogel;

3. Zn: hardystonite hydrogel;

4. Cu-Zn: cuprorivaite/hardystonite composite hydrogel.


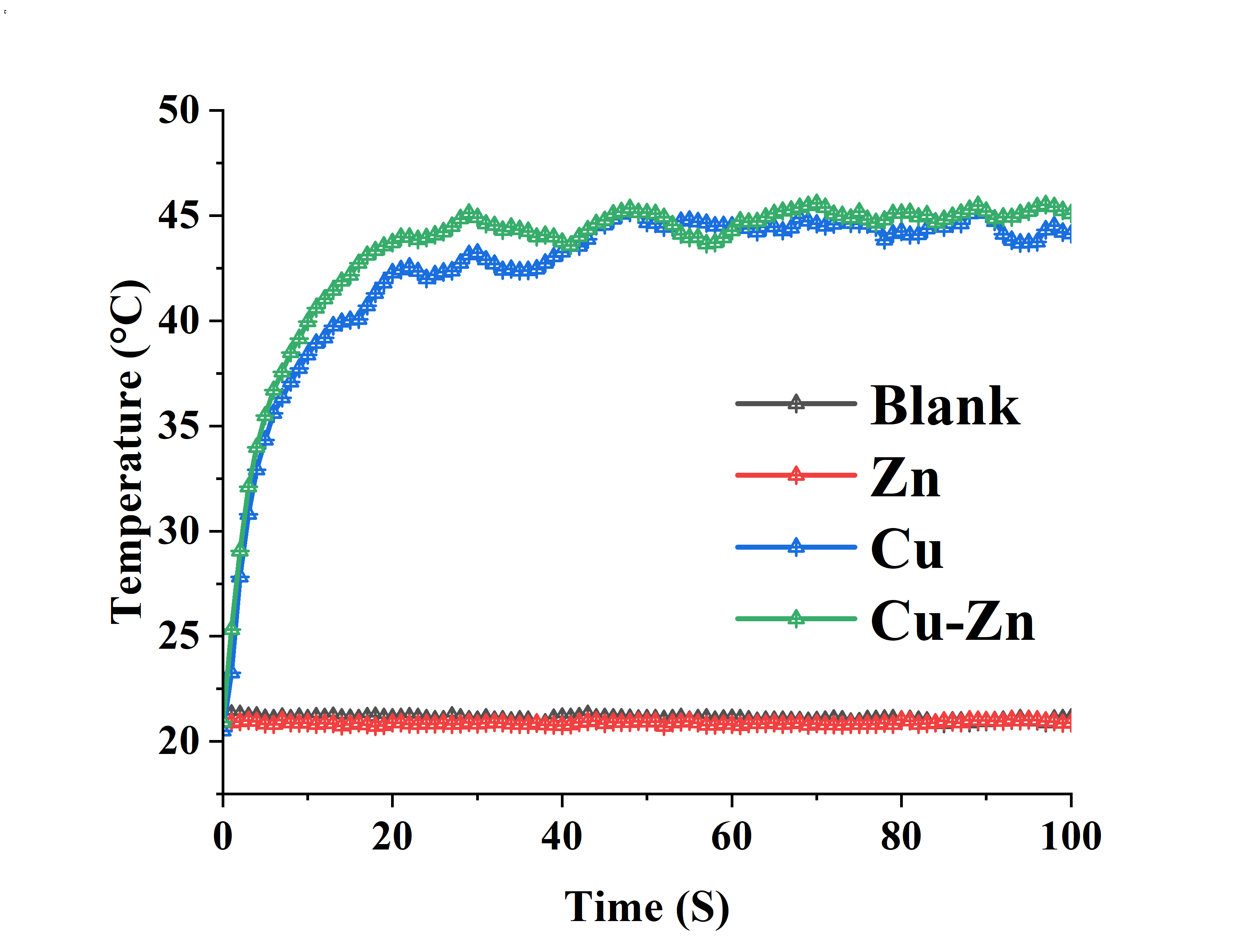


**Figure S5.** Photothermal heating curves of the composite hydrogels (808 nm, 0.30 W/cm^2^).


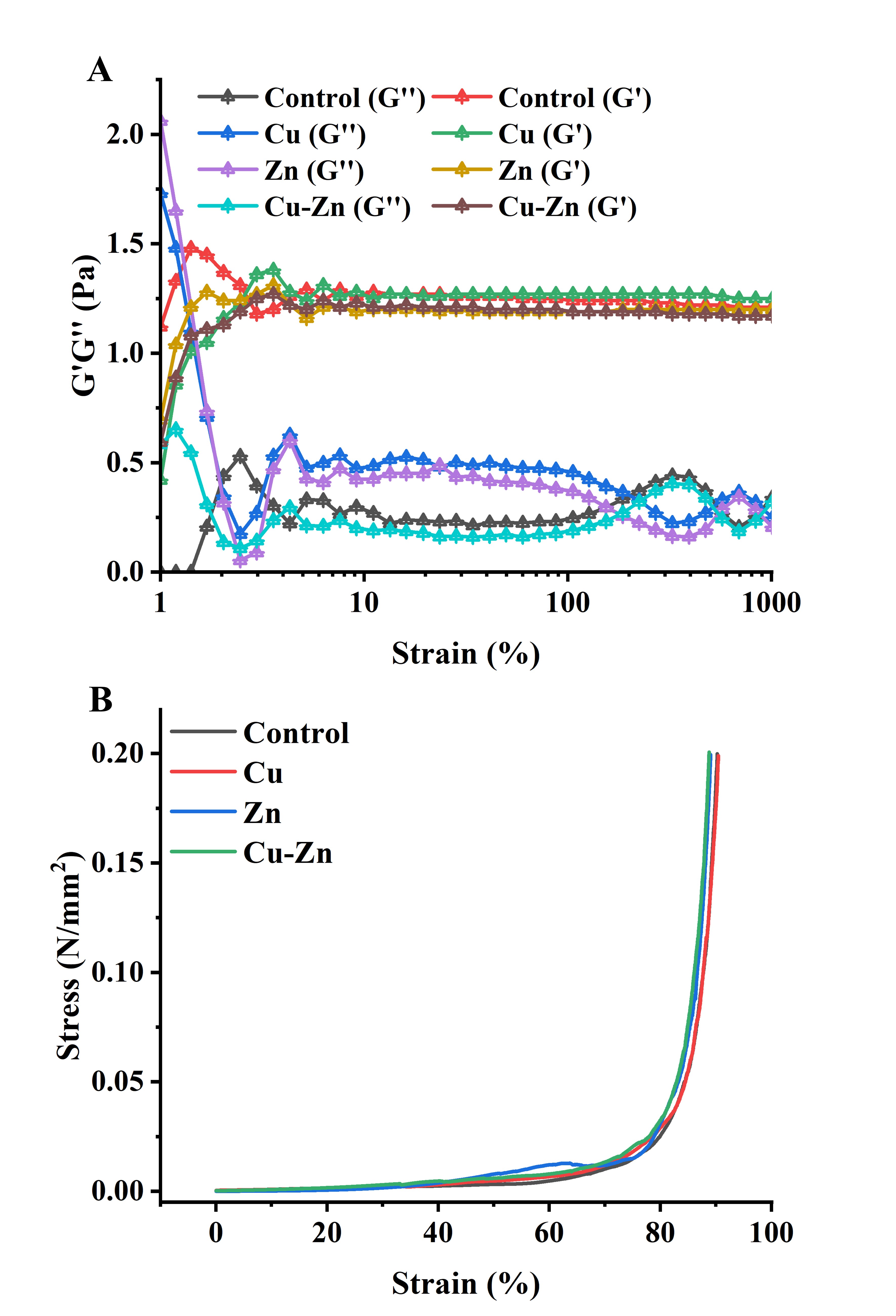


**Figure S6.** The rheological and mechanical properties of composite hydrogels. (A) The rheological properties of the composite hydrogels. (B) The stress-strain curve of the composite hydrogels.


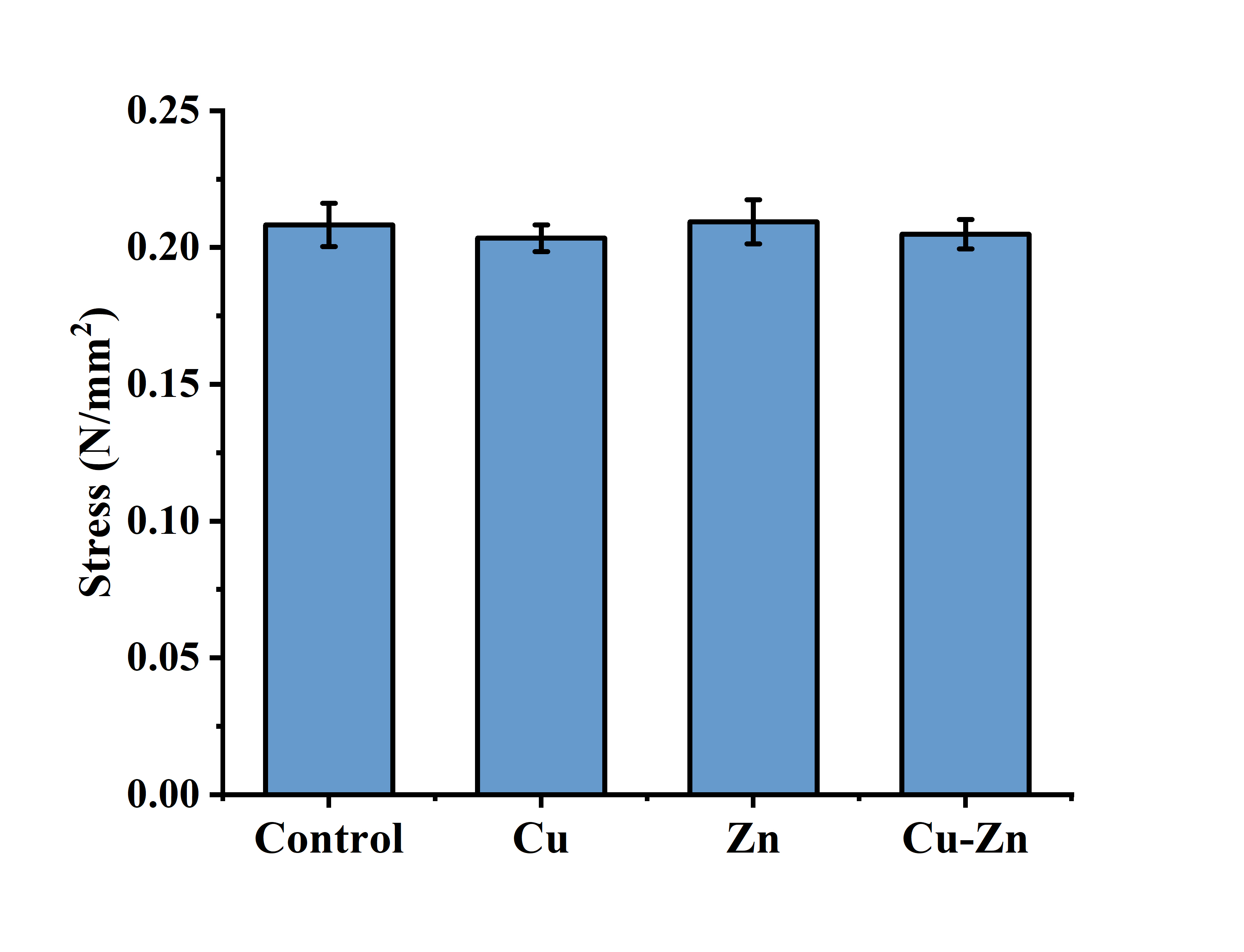


**Figure S7.** The maximum stress of the composite hydrogels.


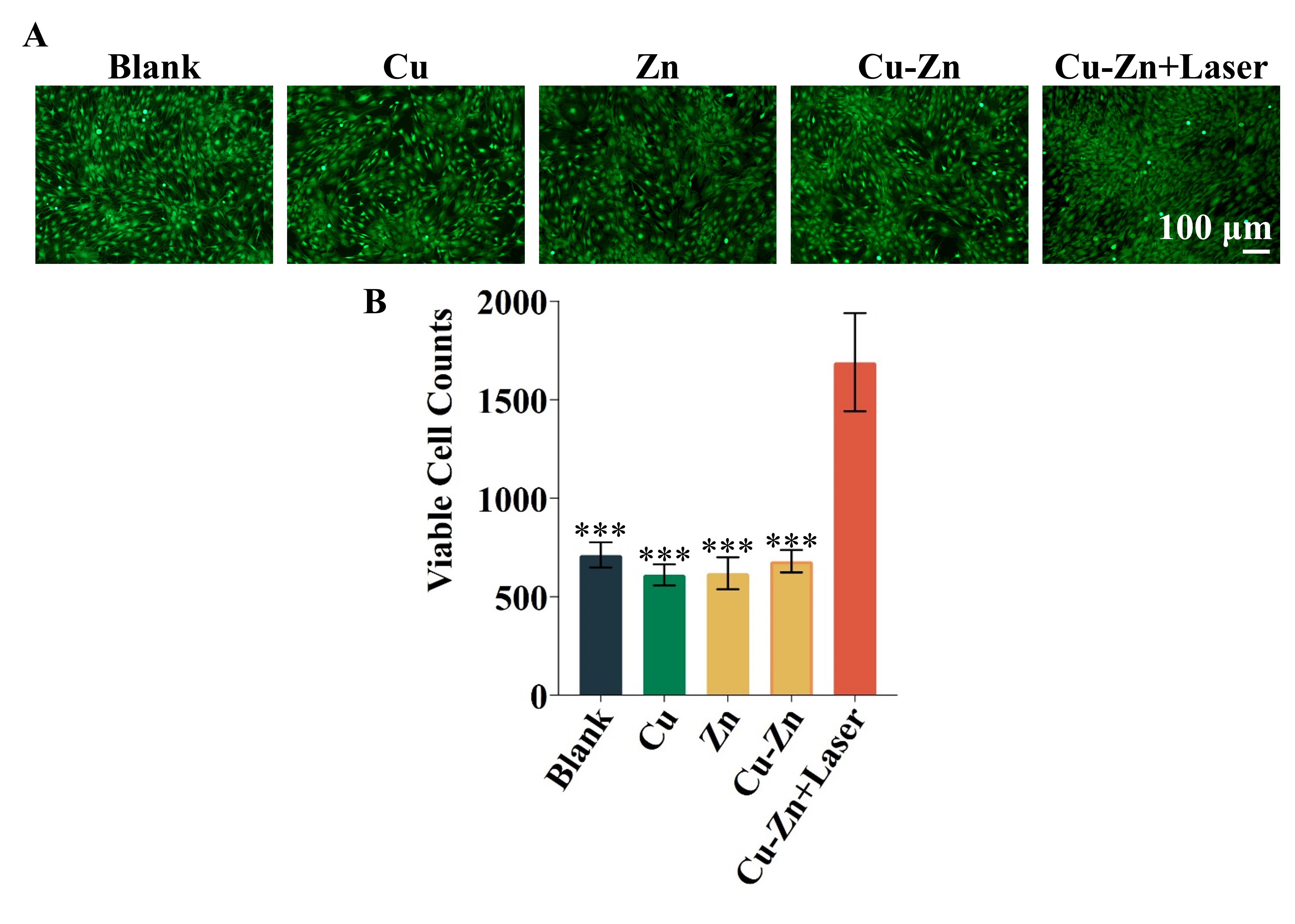


**Figure S8.** (A) Representative images of the live-dead staining of rBMSCs treated by composite hydrogels for 24 hours. (B) Quantitative analysis of the live-dead staining of rBMSCs treated by composite hydrogels for 24 hours. (***P < 0.001 compared with Cu-Zn+Laser group)

The groups were as follows:

1. Blank: Cells without any material treatment;

2. Cu: Cells treated with Cu composite hydrogel;

3. Zn: Cells treated with Zn composite hydrogel;

4. Cu-Zn: Cells treated with Cu-Zn composite hydrogel;

5. Cu-Zn+Laser: Cells treated with Cu-Zn composite hydrogel with NIR laser irradiation.


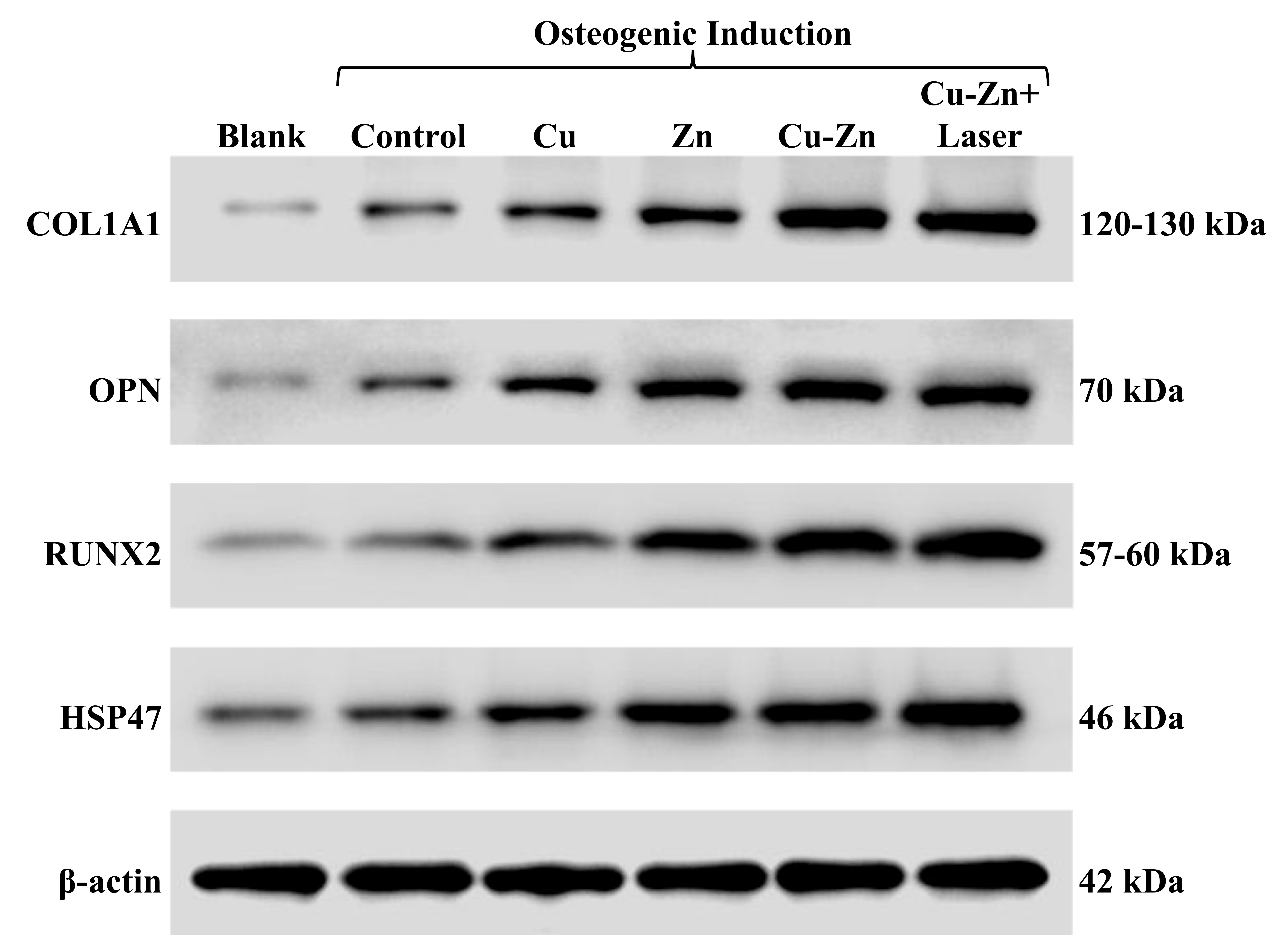


**Figure S9.** Western blot analysis of osteogenesis-related proteins (Col1A1, OPN, and RunX2) and heat shock proteins (HSP47) in rBMSCs stimulated by composite hydrogels for 14 days *in vitro*.

The groups were as follows:

1. Blank: Cells without any treatment.

Treated with osteogenic differentiation inducers:

2. Control: Cells treated with dexamethasone but without any material treatment;

3. Cu: Cells treated with dexamethasone and Cu composite hydrogel;

4. Zn: Cells treated with dexamethasone and Zn composite hydrogel;

5. Cu-Zn: Cells treated with dexamethasone and Cu-Zn composite hydrogel;

6. Cu-Zn+Laser: Cells treated with dexamethasone and Cu-Zn composite hydrogel with NIR laser irradiation.

**
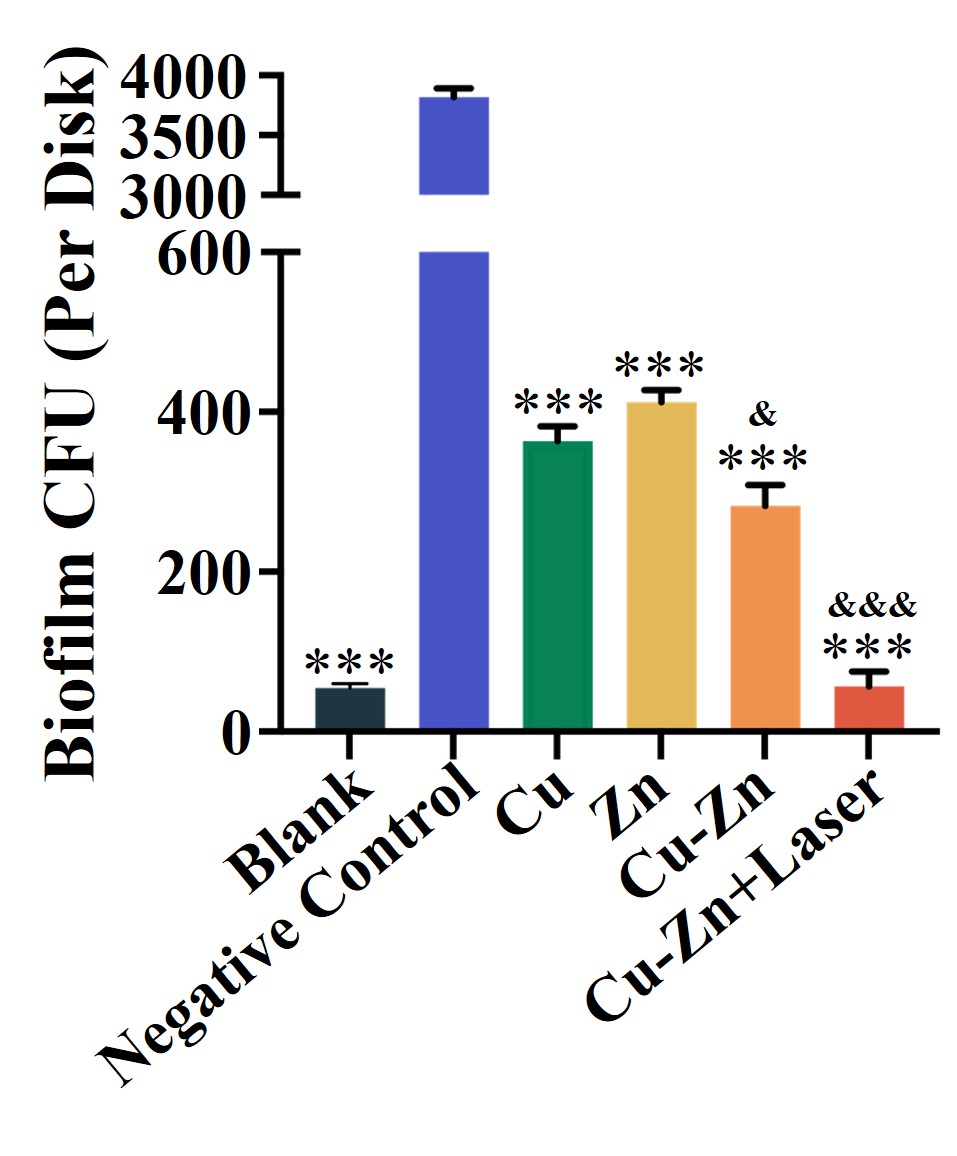
**

**Figure S10.** Quantitative analysis of the inhibition rate of bacterial around the implants by the composite hydrogel in vivo after 18 days.

(***P < 0.001 compared with Blank group; &P < 0.05, &&&P < 0.001 compared with Cu group and Zn group).

The groups were as follows:

1. Blank group: Implants not infected by periodontal pathogens;

2. Negative control group: Implants infected by periodontal pathogens and treated with Control hydrogel;

3. Cu group: Implants infected by periodontal pathogens and treated with Cu hydrogel;

4. Zn group: Implants infected by periodontal pathogens and treated with Zn hydrogel;

5. Cu-Zn group: Implants infected by periodontal pathogens and treated with Cu-Zn hydrogel;

6. Cu-Zn+Laser group: Implants infected by periodontal pathogens and treated with Cu-Zn hydrogel with NIR laser irradiation.

**
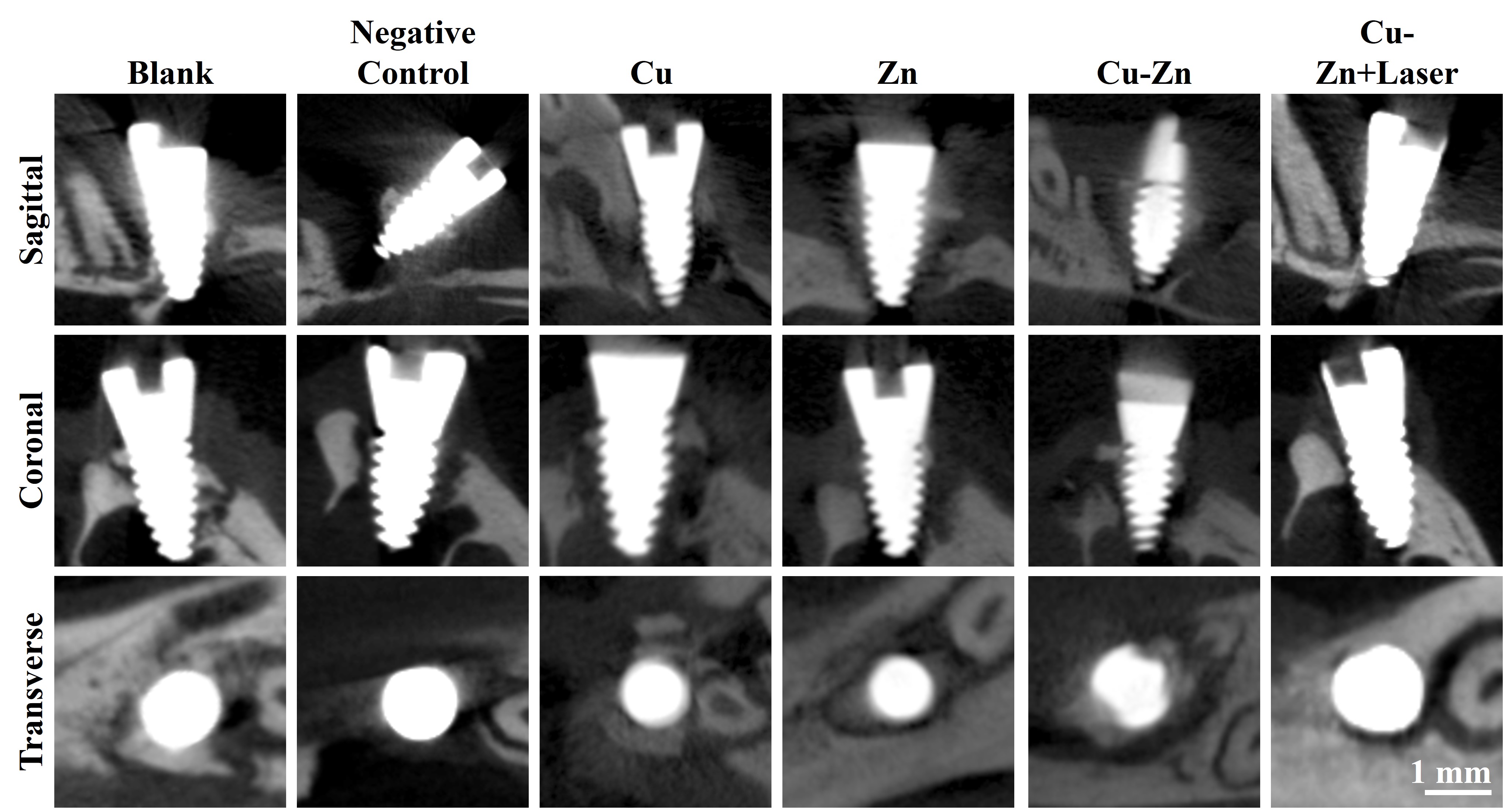
**

**Figure S11.** Micro-CT images of the implants after 18 days illustrate the osseointegration of maxillary implants from different sections (Enlarged).

The groups were as follows:

1. Blank group: Implants not infected by periodontal pathogens;

2. Negative control group: Implants infected by periodontal pathogens and treated with Control hydrogel;

3. Cu group: Implants infected by periodontal pathogens and treated with Cu hydrogel;

4. Zn group: Implants infected by periodontal pathogens and treated with Zn hydrogel;

5. Cu-Zn group: Implants infected by periodontal pathogens and treated with Cu-Zn hydrogel;

6. Cu-Zn+Laser group: Implants infected by periodontal pathogens and treated with Cu-Zn hydrogel with NIR laser irradiation.

**Table S1.** The primer sequences.

| Gene | Primer sequence (5’→3’) | |
| --- | --- | --- |
|  | Forward | Reverse |
| *ALP* | GAA AGA GAA AGA CCC CAG TTA C | ATA CCA TCT CCC AGG AAC AT |
| *BMP2* | CGT CAA GCC AAA CAC AAA CA | AGT CAT TCC ACC CCA CAT CA |
| *COL1A1* | CGT GGA AAC CTG ATG TAT GCT | ACT CCT ATG ACT TCT GCG TCT G |
| *OCN* | AAA GCC CAG CGA CTC TGA | CTC CAA GTC CAT TGT TGA GGT |
| *OPN* | CGC ATT ACA GCA AAC ACT CAG | GTC ATC GTC GTC GTC ATC AT |
| *RUNX2* | CGA AAT GCC TCT GCT GTT AT | CGT TAT GGT CAA AGT GAA ACT CT |
| *HSP47* | TAG ACA ACC GTG GCT TCA T | TCT CCT TCT CGT CGT CAT AGT A |
| *HSP70* | AGA CCT CCC TTT GAG TAT TGA | GTC CAA GAT GCT ACG AAG TG |
| *β-actin* | CCT CTA TGC CAA CAC AGT | AGC CAC CAA TCC ACA CAG |

**Table S2.** The concentration of Cu^2+^ ion released from composite hydrogels.

| The concentration of Cu^2+^ released from composite hydrogels (μg/mL). | | | | | |
| --- | --- | --- | --- | --- | --- |
| Day 1 | | | | | |
| Control | 0.03 | 0.04 | 0.04 | 0.04 | 0.04 |
| Cu | 7.33 | 8.09 | 6.54 | 9.09 | 9.28 |
| Zn | 0.03 | 0.04 | 0.04 | 0.03 | 0.04 |
| Cu-Zn | 6.76 | 8.57 | 8.74 | 9.41 | 7.38 |
| Day 2 | | | | | |
| Control | 0.03 | 0.03 | 0.03 | 0.03 | 0.03 |
| Cu | 4.41 | 4.60 | 3.77 | 4.22 | 4.15 |
| Zn | 0.04 | 0.03 | 0.04 | 0.04 | 0.03 |
| Cu-Zn | 3.52 | 4.54 | 4.64 | 4.28 | 3.95 |
| Day 3 | | | | | |
| Control | 0.03 | 0.03 | 0.03 | 0.04 | 0.03 |
| Cu | 1.83 | 1.75 | 2.14 | 2.01 | 1.97 |
| Zn | 0.04 | 0.03 | 0.03 | 0.03 | 0.04 |
| Cu-Zn | 1.75 | 2.14 | 1.86 | 1.64 | 1.81 |
| Day 4 | | | | | |
| Control | 0.04 | 0.04 | 0.03 | 0.03 | 0.04 |
| Cu | 1.09 | 1.04 | 1.04 | 1.00 | 1.08 |
| Zn | 0.03 | 0.04 | 0.03 | 0.04 | 0.04 |
| Cu-Zn | 0.89 | 1.01 | 0.95 | 0.89 | 0.89 |
| Day 5 | | | | | |
| Control | 0.04 | 0.04 | 0.04 | 0.04 | 0.04 |
| Cu | 0.56 | 0.43 | 0.47 | 0.45 | 0.51 |
| Zn | 0.04 | 0.03 | 0.03 | 0.03 | 0.03 |
| Cu-Zn | 0.57 | 0.57 | 0.42 | 0.46 | 0.48 |

**Table S3.** The concentration of Zn^2+^ ion released from composite hydrogels.

| The concentration of Zn^2+^ released from composite hydrogels (μg/mL). | | | | | |
| --- | --- | --- | --- | --- | --- |
| Day 1 | | | | | |
| Control | 0.04 | 0.03 | 0.04 | 0.04 | 0.04 |
| Cu | 0.03 | 0.04 | 0.04 | 0.04 | 0.04 |
| Zn | 9.37 | 7.24 | 7.39 | 7.48 | 8.39 |
| Cu-Zn | 8.10 | 9.42 | 7.67 | 8.13 | 9.39 |
| Day 2 | | | | | |
| Control | 0.03 | 0.03 | 0.04 | 0.03 | 0.04 |
| Cu | 0.04 | 0.04 | 0.03 | 0.04 | 0.04 |
| Zn | 4.72 | 4.64 | 3.83 | 3.51 | 4.51 |
| Cu-Zn | 4.07 | 4.69 | 3.53 | 3.55 | 4.53 |
| Day 3 | | | | | |
| Control | 0.04 | 0.04 | 0.04 | 0.04 | 0.03 |
| Cu | 0.04 | 0.03 | 0.04 | 0.03 | 0.03 |
| Zn | 2.01 | 2.36 | 2.36 | 2.23 | 2.22 |
| Cu-Zn | 1.83 | 2.11 | 2.29 | 2.23 | 2.03 |
| Day 4 | | | | | |
| Control | 0.03 | 0.03 | 0.03 | 0.03 | 0.04 |
| Cu | 0.04 | 0.03 | 0.04 | 0.04 | 0.04 |
| Zn | 0.91 | 0.90 | 0.98 | 0.97 | 0.99 |
| Cu-Zn | 1.06 | 1.15 | 0.88 | 1.10 | 1.14 |
| Day 5 | | | | | |
| Control | 0.03 | 0.04 | 0.03 | 0.04 | 0.04 |
| Cu | 0.03 | 0.03 | 0.03 | 0.03 | 0.03 |
| Zn | 0.51 | 0.53 | 0.51 | 0.59 | 0.54 |
| Cu-Zn | 0.47 | 0.57 | 0.47 | 0.44 | 0.58 |

**Table S4.** The concentration of SiO_3_^2-^ ion released from composite hydrogels.

| The concentration of SiO_3_^2-^ released from composite hydrogels (μg/mL). | | | | | |
| --- | --- | --- | --- | --- | --- |
| Day 1 | | | | | |
| Control | 0.03 | 0.04 | 0.04 | 0.03 | 0.03 |
| Cu | 8.80 | 8.44 | 7.92 | 7.26 | 11.72 |
| Zn | 9.26 | 8.01 | 12.56 | 7.99 | 7.94 |
| Cu-Zn | 14.93 | 15.47 | 19.68 | 16.10 | 14.13 |
| Day 2 | | | | | |
| Control | 0.03 | 0.04 | 0.03 | 0.04 | 0.04 |
| Cu | 4.33 | 4.22 | 4.79 | 3.90 | 4.30 |
| Zn | 4.17 | 4.31 | 4.33 | 4.18 | 3.61 |
| Cu-Zn | 8.36 | 7.47 | 9.29 | 8.63 | 8.59 |
| Day 3 | | | | | |
| Control | 0.04 | 0.04 | 0.03 | 0.04 | 0.03 |
| Cu | 1.76 | 1.84 | 2.16 | 1.92 | 1.97 |
| Zn | 2.14 | 1.95 | 1.76 | 1.82 | 2.09 |
| Cu-Zn | 4.76 | 4.46 | 4.55 | 4.99 | 4.48 |
| Day 4 | | | | | |
| Control | 0.03 | 0.04 | 0.03 | 0.03 | 0.04 |
| Cu | 1.18 | 1.19 | 1.01 | 1.11 | 1.21 |
| Zn | 1.16 | 1.24 | 0.90 | 1.06 | 0.88 |
| Cu-Zn | 2.18 | 2.41 | 1.75 | 2.37 | 2.11 |
| Day 5 | | | | | |
| Control | 0.04 | 0.03 | 0.03 | 0.04 | 0.04 |
| Cu | 0.44 | 0.53 | 0.54 | 0.52 | 0.54 |
| Zn | 0.52 | 0.58 | 0.61 | 0.50 | 0.52 |
| Cu-Zn | 1.08 | 0.99 | 0.94 | 1.00 | 1.01 |

**Video S1.** The injectable properties of composite hydrogels.

**Supplementary Experimental Section**

**Preparation of Cuprorivaite (CaCuSi_4_O_10_) and Hardystonite (Ca_2_ZnSi_2_O_7_) Bioceramics**

The Cuprorivaite bioceramics were prepared as follows: Initially, TEOS, H_2_O, and a 2 mol/L HNO_3_ solution were combined in a molar ratio of 1:8:0.16 and stirred for 30 minutes. Subsequently, following the Ca:Cu:Si stoichiometric ratio of 1:1:4, calcium nitrate and copper nitrate were carefully weighed and sequentially added to the mixture, which was then stirred at room temperature for 5 hours. After completion of the reaction, the solution was aged in a 60°C oven for 24 hours, followed by drying at 120°C for 48 hours. The resulting material was subjected to ball milling, passed through a 200-mesh sieve, and calcined at 1000°C for 3 hours. Once calcination was finalized, the material underwent further ball milling and was passed through a 400-mesh sieve to yield Cuprorivaite bioceramics.

The Hardystonite bioceramics were prepared as follows: TEOS, H_2_O, and a 2 mol/L HNO_3_ solution were mixed in a molar ratio of 1:8:0.16 and stirred for 30 minutes. Subsequently, zinc nitrate and calcium nitrate were introduced into the solution and stirred for 5 hours, maintaining a Si:Zn:Ca ratio of 2:1:2. The combined solution was then aged in a 60°C oven for 24 hours and subsequently dried at 120°C for 48 hours. The resulting dried gel was subjected to ball milling, passed through a 200-mesh sieve, and subjected to calcination at 1200°C for 3 hours. After the calcination, the material was once again ball milled and passed through a 400-mesh sieve to obtain Hardystonite bioceramics.

**Rat Bone Marrow Mesenchymal Stem Cell Culturing**

Rat bone marrow mesenchymal stem cells (rBMSCs) were derived through the whole bone marrow adherent method from two-week-old SPF grade SD rats. The process followed these steps: SD rats were euthanized via neck dislocation and then immersed in 75% ethanol for 10 minutes. In sterile conditions, the bilateral femurs and tibias were isolated, and the contents of the bone marrow cavity were flushed using MEM-α medium (Basal Media, L560KJ, China) drawn into a syringe. This mixture was subsequently centrifuged at 1000 r/min for 5 minutes, and the resulting supernatant was discarded. Following this, 3 mL of complete medium—α-MEM medium containing 10% fetal bovine serum (Gibco, 26140-079, USA), and 2% penicillin and streptomycin (Gibco, 15140-122, USA)—was added and evenly distributed. The cells were then introduced into a 6 cm diameter culture dish and placed within a cell culture incubator set at 37°C with 5% CO_2_ and 100% humidity. After the primary cell culture of 48 hours, the medium was replaced every 3 days. Once the cells reached an 80% confluence, they were detached and passaged using 0.25% trypsin (Hyclone, Canada). The ensuing generations of rBMMSCs were utilized for subsequent studies on cell viability.

**Western Blot Analysis**

Subsequent to 14 days of osteogenic differentiation, the full medium was removed, and PBS was employed to conduct 2-3 rounds of washing. To each group, 200 μL of RIPA lysate supplemented with 1% PMSF was added, followed by gentle agitation on ice for 10 minutes. The cells were then carefully detached and transferred into centrifuge tubes placed on ice for a 30-minute interval. Subsequent to centrifugation at 12000 r/min for 15 minutes at 4°C, the supernatant was harvested. The concentration of the sample was determined following the procedural guidelines of the BCA protein quantification kit (DXWB010).

Proteins were separated using sodium dodecyl sulfate-polyacrylamide gel electrophoresis, then transferred to PVDF membranes. Subsequent to blocking with a 5% skim milk powder solution at room temperature for an hour, the primary antibodies, including COL1A1 (Proteintech, 67288-1- Ig, 1:2000), OPN (Proteintech, 22952-1-AP, 1:2000), RUNX2 (Proteintech, 20700-1-AP, 1:500), HSP47 (Proteintech, 10875-1-AP, 1:2000 ), and β-actin (Proteintech, 66009-I-Ig, 1:2000), were introduced in a diluted form. The incubation was conducted overnight at 4°C. Following the incubation, the diluted HRP-labeled secondary antibody (1:5000) was promptly introduced, and the incubation was maintained at room temperature for 2 hours. After discarding the secondary antibody and conducting a thorough wash, the ECL luminescent solution was applied. Subsequent to this step, exposure and imaging were accomplished using a chemiluminescence gel imaging system.

**Immunofluorescence staining**

After the rat maxilla underwent decalcification using 10% EDTA, the implants were extracted and paraffin-embedded. Each specimen was sectioned into mesial-distal serial sections, each with a thickness of 5 μm. The paraffin sections were subjected to dewaxing and dehydration through graded alcohol, followed by antigen retrieval. Subsequently, the sections were rinsed three times with 0.01 M PBST for 5 minutes each.

After being blocked with 10% BSA in a 37°C humid chamber for 30 minutes, appropriately diluted recombinant rabbit anti-rat CD31 monoclonal antibody (ab222783, abcam, UK, diluted 1:100), mouse anti-rat CD86 monoclonal antibody (ab238468, abcam, UK, concentration of 5 µg/mL), and recombinant rabbit anti-rat CD206 polyclonal antibody (ab64693, abcam, UK, concentration of 1 µg/mL) were added. The sections were placed in a humid box at 37°C and incubated in darkness for 30 minutes. The solution was then discarded, and the cells were washed thrice with PBS for 5 minutes each time. Corresponding diluted fluorescently labeled secondary antibodies were added: goat anti-rabbit IgG H&L Alexa Fluor® 488 (ab150077, abcam, UK, diluted 1:1000) , goat anti-rabbit IgG H&L Alexa Fluor® 488 and mouse IgG H&L Alexa Fluor® 647 (ab150113, abcam, UK, diluted 1:1000) , and donkey anti-rabbit IgG H&L Alexa Fluor® 647 (ab150075, abcam, UK, diluted 1:1000).

Sections were incubated in darkness for 1 hour, followed by discarding the secondary antibody solution and washing with PBS in darkness thrice for 5 minutes each time. Subsequently, the sections were incubated with 1 μg/mL DAPI (ab104139, abcam, UK) for 1 minute, rinsed with PBS, mounted, and observed using a laser confocal microscope (LEICA TCS SP2, Leica, Germany) for photography.

**ELISA**

The secretion of inflammatory factors TNF-α, IL-1β, and IL-6 in the collected gingival crevicular fluid and serum was detected using the enzyme-linked immunosorbent assay (ELISA). The detection procedure followed the instructions of the ELISA kit (Cat: #RTA00, #RLB00, #DY506, R&D systems, USA). The summarized steps are as follows: Standard solution and test samples were prepared to establish the standard curve. The samples were diluted 1:10 with distilled water, added to the reaction plate, and incubated for 40 minutes at 37°C. After washing 4-6 times, the primary antibody working solution was added and mixed. The mixture was allowed to stand at 37°C for 20 minutes, followed by thorough washing. The enzyme-labeled antibody working solution was added, mixed well, and incubated at 37°C for 10 minutes. After thorough washing, the substrate working solution was added and the reaction was carried out in a dark place at 37°C for 15 minutes. Subsequently, the addition of the stopping solution was mixed evenly, and the absorbance value at 450 nm was measured using a microplate reader (Bio-Tek Instruments, Winooski, VT, USA). The content of the corresponding inflammatory factors was calculated using the standard curve.
